# Regulation of hepatic lipogenesis by the zinc finger protein Zbtb20

**DOI:** 10.1038/ncomms14824

**Published:** 2017-03-22

**Authors:** Gan Liu, Luting Zhou, Hai Zhang, Rong Chen, Ye Zhang, Ling Li, Jun-Yu Lu, Hui Jiang, Dong Liu, Shasha Qi, Ying-Ming Jiang, Kai Yin, Zhifang Xie, Yuguang Shi, Yong Liu, Xuetao Cao, Yu-Xia Chen, Dajin Zou, Weiping J. Zhang

**Affiliations:** 1 Department of Pathophysiology, Second Military Medical University, Shanghai 200433, China; 2 Obesity & Diabetes Research Center, Second Military Medical University, Shanghai 200433, China; 3 Department of Endocrinology, Changhai Hospital, Shanghai 200433, China; 4 National Key Laboratory of Medical Immunology, Second Military Medical University, Shanghai 200433, China; 5 Institute of Immunology, Second Military Medical University, Shanghai 200433, China; 6 Department of General Surgery, Changhai Hospital, Shanghai 200433, China; 7 Barshop Institute for Longevity and Aging Studies, University of Texas Health Science Center at San Antonio, San Antonio, Texas 78245, USA; 8 Institute of Nutritional Sciences, Shanghai Institute of Biological Sciences, Chinese Academy of Science, Shanghai 200031, China

## Abstract

Hepatic *de novo* lipogenesis (DNL) converts carbohydrates into triglycerides and is known to influence systemic lipid homoeostasis. Here, we demonstrate that the zinc finger protein Zbtb20 is required for DNL. Mice lacking *Zbtb20* in the liver exhibit hypolipidemia and reduced levels of liver triglycerides, along with impaired hepatic lipogenesis. The expression of genes involved in glycolysis and DNL, including that of two ChREBP isoforms, is decreased in livers of knockout mice. Zbtb20 binds to and enhances the activity of the *ChREBP-α* promoter, suggesting that altered metabolic gene expression is mainly driven by ChREBP. In addition, ChREBP-β overexpression largely restores hepatic expression of genes involved in glucose and lipid metabolism, and increases plasma and liver triglyceride levels in knockout mice. Finally, we show that Zbtb20 ablation protects from diet-induced liver steatosis and improves hepatic insulin resistance. We suggest ZBTB20 is an essential regulator of hepatic lipogenesis and may be a therapeutic target for the treatment of fatty liver disease.

## Abstract (web summary)

*De novo* lipogenesis is tightly controlled by hormonal and nutritional signals and plays an important role in energy homoeostasis. Here, Liu *et al*. show that zinc finger protein ZBTB20 regulates the expression of key glycolytic and lipogenic genes by modulating ChREBP expression and transcriptional activity.

In mammals, the liver is the major site of carbohydrate metabolism and triglyceride synthesis. Upon ingestion of excess carbohydrates, the liver converts the carbohydrates into triglycerides (TG) by *de novo* lipogenesis (DNL), packages them in very low density lipoprotein (VLDL) and secretes them into plasma for energy storage in adipose tissue. Coordinately regulated by hormones (for example, insulin and glucagon) and nutrients, hepatic lipid metabolism plays a critical role in glucose, lipid and energy homoeostasis, the dysregulation of which leads to fatty liver, insulin resistance and atherosclerosis. Of particular importance, elevated DNL in liver contributes to the development of non-alcohol fatty liver disease (NFALD) in rodents and humans^1^,^2^,^3^,^4^.

DNL is controlled by a highly dynamic transcriptional regulatory network, which mainly involves sterol response element binding protein-1c (SREBP-1c) and carbohydrate response element binding protein (ChREBP)^1^. In response to different hormonal and nutritional signals, these two factors regulate distinct key glycolytic enzyme genes. SREBP-1c is required to induce glucokinase (GCK) by insulin, while ChREBP is essential for the induction of pyruvate kinase liver and red blood cell (PKLR) in response to glucose^5^,^6^. On the other hand, they act in synergy to induce the critical lipogenic genes encoding fatty acid synthase (Fasn), elongation of very long chain fatty acids protein 6 (ELOVL6) and stearyl coenzyme A desaturase 1 (SCD1)^7^. As a result, ablation or inhibition of SREBP1c or ChREBP partially impairs lipid synthesis and improves hepatic steatosis^8^,^9^,^10^,^11^. For example, liver-specific deletion of SREBP cleavage-activating protein (SCAP), which is an escort protein mediating SREBP1 translocation to Golgi apparatus and subsequent activation, leads to a decline in plasma lipid levels and liver triglyceride contents in the mice, as a result of reduced hepatic DNL^12^. These results suggest that manipulation of the regulatory hierarchy of DNL might be of pharmacological interest to treat NAFLD.

ChREBP has two isoforms due to distinct promoter-driven transcription initiation and alternative splicing, which exhibit distinctive features in protein structure, intracellular location, transcriptional activity and expression pattern^13^. Structurally, ChREBP-β is a truncated form of ChREBP-α, with 177 amino acids lost at the N terminal. Functionally, ChREBP-β can be regarded as a constitutive active form of ChREBP, and plays a critical role in the regulation of DNL and metabolic homoeostasis^13^,^14^. ChREBP-α protein shuttles between cytoplasm and nucleus depending on its functional states, while ChREBP-β seems to be a nuclear protein. Despite of its more potent transcriptional activity, ChREBP-β is expressed at much lower levels relative to ChREBP-α, such that it is hard to be detected in liver or white adipose tissue using the currently available antibodies. Intriguingly, there is a carbohydrate response element (ChoRE) site in ChREBP-β gene promoter, allowing it to be positively regulated by both isoforms, which presumably serves as an auto-regulatory mechanism of signal amplification and positive feedback in response to carbohydrate stimulation. Therefore, ChREBP-β is one of ChREBP-α target genes^13^. Both isoforms need Max-like protein (Mlx) as a obligate heteromeric partner for the transcriptional activity^13^,^15^.

The expression and activity of ChREBP are tightly regulated by glucose in liver^16^. At the posttranslational level, glucose modulates ChREBP phosphorylation and acetylation, thus controlling its entry into nucleus and transcriptional activity^17^,^18^. At the transcriptional level, ChREBP-α is activated by glucose^6^,^7^, probably through its metabolites^19^,^20^. Notably, ChREBP-α has been identified as a direct target gene of liver X receptor (LXR) and thyroid hormone receptor β (TRβ) in liver^21^,^22^,^23^, which are nuclear receptors involved in the transcriptional regulation of carbohydrate and lipid metabolism^24^,^25^,^26^. However, both of them are not necessary for the induction of ChREBP expression by glucose or refeeding^6^,^23^. Therefore, the molecular mechanism about the regulation of ChREBP transcription is still largely unknown.

Zinc finger and BTB domain-containing protein 20 (ZBTB20, also known as DPZF) belongs to a subfamily of zinc finger proteins containing C2H2 Krüppel-type zinc fingers and BTB/POZ domain^27^. It is implicated that ZBTB20 may play an important role in glucose and lipid homoeostasis^28^. The mice lacking Zbtb20 exhibit hypoglycemia, depletion of energy stores, insulin hypersensitivity, growth retardation and premature lethality. Given its high expression in adult liver^29^, in this study, we characterized the metabolic functions of hepatic Zbtb20 using its liver-specific knockout mice, which exhibit hypolipidemia and impaired hepatic lipogenesis. Mechanistically, Zbtb20 undergoes nuclear translocation from cytosol in response to glucose stimulation, then binds to and activates the transcription of ChREBP-α promoter, thereby modulating the glycolytic and lipogenic enzyme genes largely in a ChREBP-dependent manner. Our data demonstrate that ZBTB20 is an essential regulator of lipid metabolism, and may serve as a therapeutic target for fatty liver disease.

## Results

### Disrupted lipid homoeostasis by the loss of liver Zbtb20

Our previous study has shown that Zbtb20 is highly expressed in adult liver^29^. To investigate its functional relevance, we have generated the mice lacking *Zbtb20* specifically in the liver (LZB20KO) using *alb* promoter-driven Cre transgenic mice. LZB20KO mice did not display any noticeable gross abnormalities, and had apparently normal liver mass and architecture, with no obvious sign of liver injury as evidenced by normal serum levels of alanine aminotransferase (ALT)^29^ (Supplementary Table 1). When fed the standard chow diet, LZB20KO mice did not show significant difference in body weight, lean mass or fat mass compared with their gender and age-matched control. Moreover, their food intake and energy expenditure measured by indirect calorimetry were comparable to control group (Supplementary Fig. 1). In addition, plasma glucose levels in LZB20KO mice were unchanged in the fasted state, but slightly and significantly decreased in the fed state compared to control, whereas plasma insulin levels were similar between the two groups in either fasted or fed state (Supplementary Table 1). Glucose tolerance test and insulin tolerance test showed that LZB20KO mice had normal glucose clearance and insulin sensitivity (Supplementary Fig. 2a,b). These data suggest that liver Zbtb20 is largely dispensable for glucose homoeostasis under physiological condition, and the severe hypoglycaemia phenotype previously observed in Zbtb20 global knockout mice^28^ is most likely due to its deficiency in extra-hepatic tissues.

**Figure 1:**
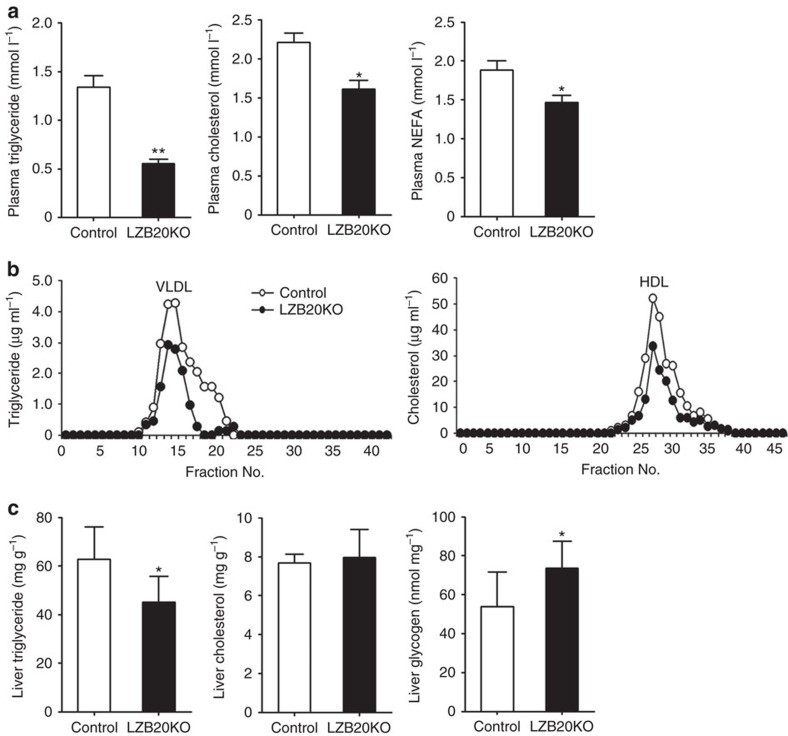
Diminished plasma and hepatic lipid levels in liver-specific Zbtb20 knockout mice. Male wild-type or LZB20KO mice at the age of 3–4 months were fed on normal chow. (**a**) Plasma levels of TG, cholesterol and free fatty acid in the fasted state (*n*=20 for TG, *n*=12 for TC and FFA)). (**b**) Distribution of plasma triglyceride and cholesterol was determined by FPLC separation of lipoprotein particles. (**c**) Liver contents of TG, cholesterol and glycogen in the fed state. Data are represented as mean±s.e.m. *P<0.05; **P<0.01 versus control (Student's *t*-test).

Interestingly, fasted plasma TG levels were 60% lower in LZB20KO mice than that of control group, and to some less extent, their plasma cholesterol and free fatty acid levels were also markedly reduced (Fig. 1a). In the fasted state, plasma TG is mainly derived from hepatic secretion in the form of VLDL particles that transport TG together with cholesterol to peripheral tissues^30^. Hence, we performed fast protein liquid chromatography (FPLC) analysis on plasma lipoprotein profile. LZB20KO mice showed a marked decrease in the contents of both VLDL-TG and high-density lipoprotein (HDL)-cholesterol relative to control under the fasted condition (Fig. 1b). In contrast, plasma apolipoprotein B (apoB), a major protein component of VLDL, was maintained at similar levels in both groups (Supplementary Table 1).

Then we further examined lipid contents in the liver. LZB20KO mice had normal cholesterol contents, but showed a marked (∼30%) decrease in liver TG contents compared to control in the fasted state (Fig. 1c), indicating that their hypolipidemia was not because of the lipid retention in the liver. On the other hand, liver glycogen contents were mildly elevated in LZB20KO mice relative to control in the fed state, which is likely due to the increased production and/or inhibited glycolysis of glycogen. Taken together, these findings suggest that liver ZBTB20 plays a critical role in lipid homoeostasis, which may also involve carbohydrate metabolism.

### Zbtb20 deletion impairs hepatic lipid synthesis and secretion

**Figure 2:**
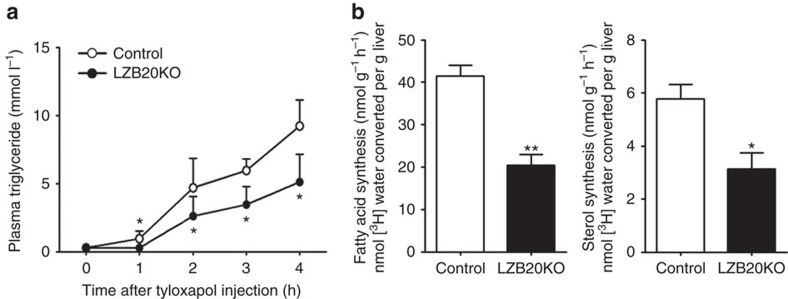
Impaired hepatic lipid synthesis and TG secretion in the absence of Zbtb20. Male control or LZB20KO mice at the age of 3–4 months were fed on normal chow. (**a**) Plasma TG levels after intraperitoneal injection of tyloxapol following a 4-h fast. *n*=4. (**b**) *In vivo* hepatic lipogenesis was determined by measuring the incorporation of ^3^H-H_2_O into fatty acids and sterols of the liver. *n*=7 per group. Data are represented as mean±s.e.m. *P<0.05; **P<0.01 versus control (Student's *t*-test).

To determine whether ZBTB20 regulates hepatic lipid synthesis and secretion, we first measured lipid secretion rate *in vivo* by intraperitoneally injecting tyloxapol into control and mutant mice, which blocks VLDL clearance by inhibiting LPL, hepatic lipase and endothelial lipase^31^. Under this condition, fasted plasma TG levels largely reflect the hepatic VLDL secretion. After tyloxapol treatment, chow-fed LZB20KO mice displayed a marked decrease in the rate of plasma TG accumulation compared with control (Fig. 2a). FPLC analysis showed that the plasma from tyloxapol-treated LZB20KO mice contained lower amount of TG in the VLDL fraction, as well as lower cholesterol contents in the VLDL and HDL fractions than that of control counterparts, while the protein contents were similar between two groups in both VLDL and HDL fractions (Supplementary Fig. 3). These indicated that ZBTB20 deficiency resulted in a decrease in hepatic secretion of TG and cholesterol, likely due to their reduced uploads onto VLDL particles prior to secretion. Furthermore, we measured DNL in the liver by injection of ^3^H-water as a tracer. LZB20KO liver displayed a substantial reduction in the synthesis of fatty acids and sterols relative to control (Fig. 2b). Taken together, these data suggest that Zbtb20 plays an essential role in hepatic lipid synthesis.

### Characterization of glycolytic and lipogenic gene expression

**Figure 3:**
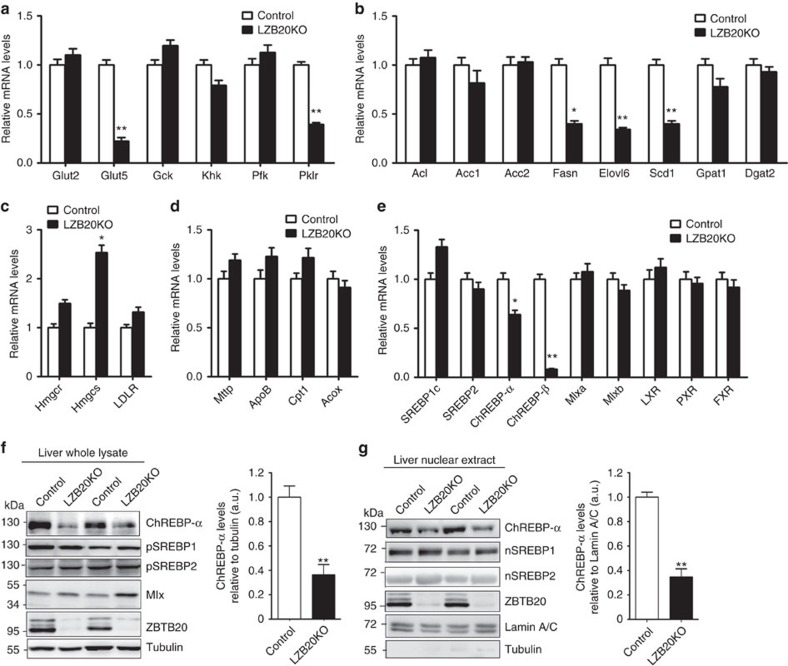
Decreased expression of the glycolytic and lipogenic genes in Zbtb20-deficient liver. Male control or LZB20KO mice at the age of 3–4 months were fed on normal chow. *n*=5 per group. (**a**–**e**) The mRNA expression levels of the genes involved in glycolysis (**a**), lipogenesis (**b**), sterol synthesis and cholesterol metabolism (**c**), TG secretion and fatty acid oxidation (**d**) and transcriptional regulators of lipid metabolism (**e**) were determined in the liver by quantitative RT-PCR with *36B4* mRNA as internal control. (**f**,**g**) Western blot analysis for protein levels of ChREBP, Mlx and SREBP in the whole lysate (**f**) and nuclear extract (**g**) of the liver. pSREBP and nSREBP indicate precursor and nuclear SREBP, respectively. Tubulin and Lamin A/C were used as internal controls for the whole lysates and nuclear extracts, respectively. A representative western blot for three independent experiments was shown with the quantification plot based on scanning densitometry analysis. Lack of cytoplasmic protein contamination was confirmed in the nuclear extracts by probing tubulin (Supplementary Fig. 13). Data are represented as mean±s.e.m. *P<0.05; **P<0.01 versus control (Student's *t*-test).

To explore the molecular mechanisms by which Zbtb20 regulates hepatic lipogenesis, we first examined the expression of glycolytic and lipogenic enzyme genes in the liver from control and LZB20KO mice on the normal chow or a high-carbohydrate diet (HCD) rich in starch and sucrose (72.2% carbohydrates, 1% fat and 26.8% protein by energy), the latter enhances endogenous hepatic lipogenesis and chronically induces hepatic steatosis. Among the genes critical for glycolysis, fructose transporter 5 (*Glut5*) and *Pklr* were decreased by 61–78% at mRNA levels in chow or HCD-fed livers compared to the control counterparts (Fig. 3a; Supplementary Table 2), while glucose transporter 2 (*Glut2*), *Gck*, fructose kinase (*Khk*) and phosphofructose kinase (*Pfk*) were not significantly affected at mRNA levels. Among the genes encoding rate-limiting lipogenic enzymes, while acetyl-CoA carboxylase 1 and 2 (*Acc1*, *Acc2*), glycerol-3-phosphate acyltransferase (*Gpat*) family members, 1-acylglycerol-3-phosphate-*O*-acyltransferase (*Agapt*) family members and diacylglycerol acetyltransferase 2 (*Dgat2*) were not significantly changed at mRNA levels in the mutants (Supplementary Table 2; Supplementary Fig. 4), the mRNA levels *of Fasn*, *Elovl6*, and *Scd1* were decreased by 42–74% in chow or HCD-fed LZB20KO liver relative to control (Fig. 3b). Of note, genetic deletion liver *Elovl6* or *Scd1* leads to profound alterations of triglyceride metabolism and/or insulin sensitivity^32^,^33^,^34^,^35^,^36^. Among the genes encoding rate-limiting enzymes of sterol synthesis, 3-hydroxy-3-methylglutaryl-CoA reductase (*Hmgcr*) mRNA levels showed a tendency of increase in mutant liver without reaching statistical significance, while 3-hydroxy-3-methylglutaryl-CoA synthase (*Hmgcs*) mRNA levels was increased compared to control (Fig. 3c). However, western blot analysis showed that either HMGCR or HMGCS was not significantly changed at the protein levels (Supplementary Fig. 5). Furthermore, Zbtb20 deletion did not significantly change the mRNA levels of carnitine palmitoyltransferase 1 (*Cpt1*) and acyl-CoA oxidase (*Acox*) in the liver, which are critical for fatty acid oxidation. In addition, the mRNA levels of TG secretion-related microsomal triglyceride transfer protein (*Mttp*) and *ApoB* were not significantly different between the two genotypes (Fig. 3d), and LDL receptor (LDLR), which mediates hepatic uptake of circulating LDL-cholesterol, was also not affected at mRNA levels (Fig. 3c).

Then we further examined expression of transcription factors which are critically involved in liver lipid metabolism. Notably, *ChREBP-α* and *ChREBP-β* mRNA expression levels were decreased by 40 and 90% in LZB20KO liver, respectively (Fig. 3e). By western blot analysis using polyclonal antibodies recognizing both isoforms (Supplementary Fig. 6), we found that ChREBP-α was the predominant form of ChREBP protein in the liver, while ChREBP-β was almost undetected due to its physiologically low expression levels. LZB20KO liver displayed a robust decrease in ChREBP-α protein levels both in the whole lysate (61%) and in the nuclear extracts (65%) under the fed condition (Fig. 3f,g). Whereas, *Mlx* genes, which encodes an obligate dimerization partner for ChREBP transcriptional activity^15^, was expressed normally at both mRNA and protein levels (Fig. 3e,f). On the other hand, SREBP1c mRNA levels were slightly increased in LZB20KO liver, but did not reach statistical significance (Fig. 3e), and its protein levels were apparently normal either in the whole lysate or in the nuclear extracts as the mature form under the fed condition (Fig. 3f), which was consistent with normal expression of its target gene *Gck* (Supplementary Table 2), the major hexokinase in the liver regulated by insulin but not glucose^37^. In addition, SREBP2, a critical regulator of sterol synthesis activated by hepatic sterol contents^38^, was not changed at mRNA or protein levels, which was consistent with the normal expression of its target genes *Hmgcr* and the normal contents of liver cholesterol. Other transcriptional factors and cofactors involved in liver lipid metabolism such as *LXR*, *RXR*, and *FXR*, *PXR PPARα*, *PPARγ*, *PGC-1α*, were not altered by the loss of Zbtb20 (Fig. 3e; Supplementary Fig. 7). Put together, these results suggest that Zbtb20 may regulate a subset of the genes involved in glycolysis and lipogenesis, including *Glut5*, *Pklr*, *Fasn*, *Elovl6*, *Scd1*, as well as the transcription factor *ChREBP*.

### Zbtb20 regulates glycolytic and lipogenic genes via ChREBP

**Figure 4:**
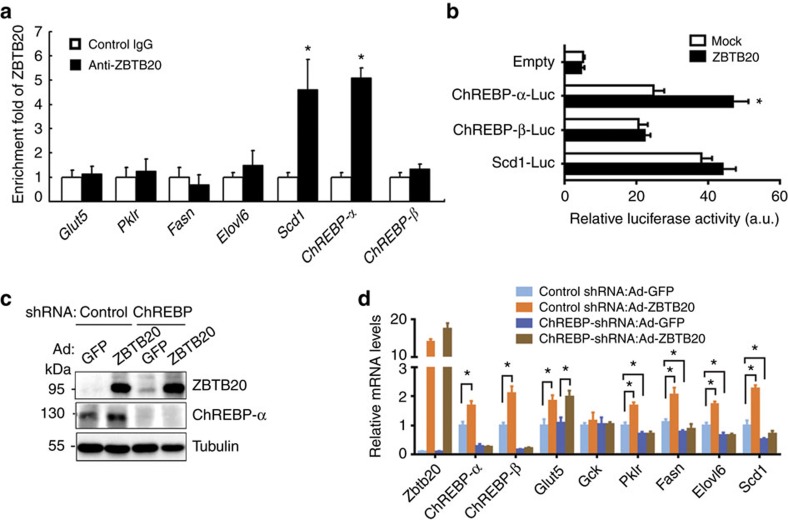
Zbtb20 regulates hepatic lipogenesis partially through ChREBP. (**a**) Zbtb20 binds to Scd1 and ChREBP-α promoters in the liver. Liver from normal chow-fed wild-type mice was subjected to ChIP analysis in the fed state. Values represent relative increase of real-time PCR signals for ChIP with anti-ZBTB20 antibodies compared to that for ChIP with control IgG. *n*=4 per group. (**b**) ZBTB20 overexpression enhanced the transcriptional activity of the ChREBP-α promoter in Zbtb20-deficient hepatocytes. The luciferase activity was detected 48 h after plasmid transfection, and normalized by the internal control RL-TK. The experiment was repeated three times independently. (**c**,**d**) ZBTB20 overexpression induced the glycolytic and lipogenic genes partially through ChREBP in LZB20KO liver. 3-4 month-old male LZB20KO mice were i.v. injected with shRNA-expressing adenoviruses together with Ad-ZBTB20 or Ad-GFP. Five days later, the gene expression was detected in the liver by western blot (**c**) and qRT-PCR (**d**). *n*=5 per group. Data are represented as mean±s.e.m. *P<0.05 versus control or indicated groups (Student's *t*-test).

To understand the mechanisms by which ZBTB20 regulates the above glycolytic and lipogenic genes, we first performed chromatin immunoprecipitation (ChIP) analysis to analyse their promoter occupancy by ZBTB20. ZBTB20 showed robust binding to the promoters of *ChREBP-α* and *Scd1* in the liver, while no significant binding was detected in the promoters of *ChREBP-β*, *Glut5*, *Pklr*, *Fasn* or *Elovl6* (Fig. 4a). Of note, ChREBPs can positively regulate the expression of *ChREBP-β*, *Glut5*, *Pklr*, *Fasn*, *Elovl6* and *Scd1* through the ChoER sites in their promoters^13^,^39^,^40^, and *Pklr* is regarded as a typical target gene of ChREBP^5^. Furthermore, luciferase reporter assays demonstrated that ZBTB20 overexpression could significantly enhance the transcriptional activity of *ChREBP-α* promoter in Zbtb20-deficient primary hepatocytes, while had no effects on that of *ChREBP-β* or *Scd1* promoter (Fig. 4b), suggesting that Zbtb20 could directly regulate *ChREBP-α* gene expression. Considering that the transcriptional activity of ChREBP protein is regulated by glucose metabolites^20^, such as glucose-6-phosphate (G6P), we measured G6P contents in the liver, and found that there was no significant change in the mutant compared with control (Supplementary Fig. 8a). Moreover, the luciferase reporter assays in 293 T cells demonstrated that Zbtb20 overexpression did not significantly affect the transcriptional activity of the *ChREBP-β* promoter or the ChoRE reporter in the presence of ChREBP-α/Mlx (Supplementary Fig. 8b,c). These results indicate that Zbtb20 may have no direct effects on the transcriptional activity of ChREBP-α protein. Thus, we reasoned that ZBTB20 might regulate the glycolytic and lipogenic genes mainly through modulating the expression of ChREBPs.

To address whether ChREBP is essential for the regulation of the above glycolytic and lipogenic genes by Zbtb20, we overexpressed ZBTB20 in LZB20KO liver by intravenous injection of ZBTB20-expressing adenoviruses (Ad-ZBTB20) or GFP-expressing adenoviruses (Ad-GFP) as control together with shRNA-expressing adenoviruses. Five days later, the mRNA expression levels of *ChREBP-*α, *ChREBP-β*, *Glut5*, *Pklr*, *Fasn*, *Scd1* and *Elovl6* were markedly increased by ZBTB20 overexpression in the mutant liver (Fig. 4c,d). Administration of adenoviruses expressing ChREBP-shRNA (Ad-ChREBP-shRNA) inhibited ChREBP-α expression at the mRNA and protein levels, as well as *ChREBP-β* mRNA levels, albeit ChREBP-β protein levels undetectable. ChREBP inhibition in LZB20KO liver led to a further decrease in the mRNA expression levels of *Pklr*, *Fasn*, *Elovl6* and *Scd1*, without significant effect on *Glut5* expression. Of note, when ChREBP expression was inhibited, ZBTB20 overexpression did not significantly alter the mRNA expression levels of *Pklr*, *Fasn*, *Elovl6* or *Scd1* in mutant liver, suggesting that ChREBP is required for their expression regulation by ZBTB20. By contrast, ChREBP inhibition did not affect *Glut5* upregulation by ZBTB20 overexpression, indicating that ZBTB20 regulates *Glut5* expression in a ChREBP-independent manner.

### ChREBP restores liver glucose and TG metabolism in knockout

**Figure 5:**
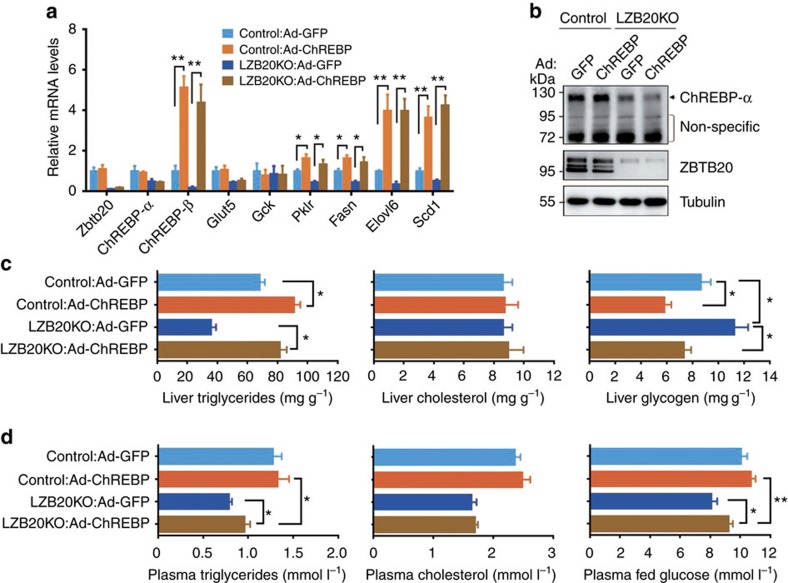
ChREBP overexpression partially restores hepatic lipogenesis. 3-4 month-old male control or LZB20KO mice were i.v. injected with ChREBP-β-expressing adenoviruses (Ad-ChREBP) or Ad-GFP as control. One week later, gene expression and phenotypes were analysed. *n*=5 per group. (**a**) Partial restoration of glycolytic and lipogenic gene expression in LZB20KO liver by ChREBP-β overexpression. (**b**) ChREBP-β protein was still undetected in ChREBP-β-overexpressed liver from by western blot. NS, nonspecific band. (**c**,**d**) ChREBP-β overexpression partially restored lipid and glucose metabolism in the plasma (**c**) and liver (**d**). Data are represented as mean±s.e.m. *P<0.05; **P<0.01 versus indicated groups (Student's *t*-test).

To further assess the potential role of ChREBP in the regulation of glucose and lipid metabolism by Zbtb20, we overexpressed the constitutively active ChREBP-β in control and LZB20KO liver by intravenous injection of ChREBP-β-expressing adenoviruses (Ad-ChREBP). One week after the administration of Ad-ChREBP, *ChREBP-β* mRNA levels were increased by 5.1-fold in the wild-type liver compared with Ad-GFP-treated control, while its protein expression was still undetectable by western blot (Fig. 5a,b), likely due to the low expression levels under detection limits. As a result, the mRNA expression levels of *Pklr*, *Fasn*, *Elovl6* and *Scd1* were significantly increased by ChREBP-β overexpression in control liver. More importantly, ChREBP-β overexpression could largely or even completely restore the mRNA levels of *Pklr*, *Fasn*, *Elovl6* and *Scd1* in mutant liver, while *Glut5 and Gck* expression was not changed (Fig. 5a). Phenotypically, ChREBP-β overexpression resulted in a significant increase in TG contents and a significant decrease in glycogen contents in control liver. More interestingly, the TG and glycogen contents were almost completely restored by ChREBP-β overexpression in mutant liver, while TC contents were not affected in either control or mutant liver (Fig. 5c). Then we further analysed glucose and lipid levels in the plasma. Compared with GFP control, ChREBP-β overexpression did not cause a significant change in plasma TG, TC or glucose levels in control mice. However, in LZB20KO mice, the plasma levels of TG and glucose were mildly but significantly increased by ChREBP-β overexpression compared with GFP control, which were still lower than those in control mice, indicating a partial restoration. Meanwhile, plasma TC levels were not significantly changed by ChREBP-β overexpression compared with GFP control in mutant mice (Fig. 5d). Collectively, these results suggest that ChREBP overexpression could largely restore glucose and TG metabolism in the liver, and to some less extent, in the plasma of Zbtb20-deficient mice.

### Carbohydrates regulate hepatic Zbtb20 nuclear translocation

**Figure 6:**
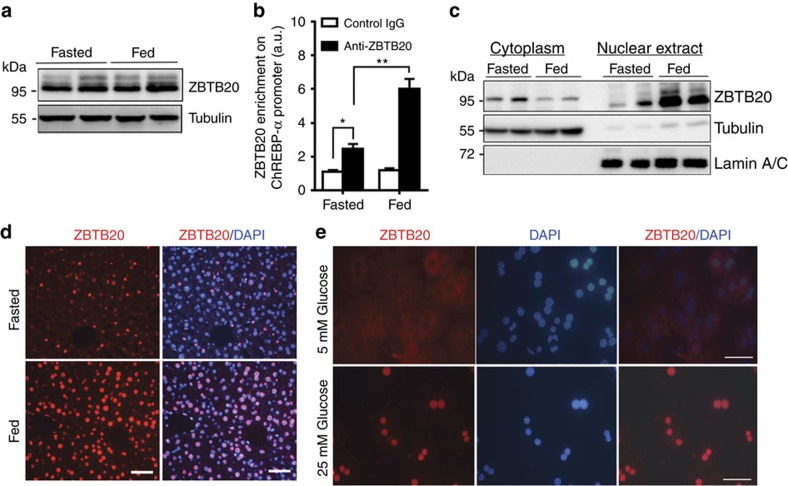
Carbohydrate regulate Zbtb20 nuclear translocation and transcriptional activity. (**a**–**d**) 3-4 month-old male C57BL/6 mice on normal chow were fasted for 16 h or in the fed state. ZBTB20 protein levels were similar between the two states in the liver whole lysates (**a**), but its binding to ChREBP-α promoter was enhanced in the fed state (**b**). Western blot (**c**) and immunohistochemical analysis (**d**) revealed an increase in hepatic nuclear localization of ZBTB20 in the fed liver compared to the fasted control. GAPDH and Lamin A/C were used as a loading control for the cytoplasmic and nuclear fraction, respectively. Scale bars, 50 μm. *n*=5 per group. (**e**) ZBTB20 nuclear translocation was regulated by glucose stimulation in primary hepatocytes. Primary hepatocytes were isolated and cultured with the medium containing 5 mM glucose for 36 h before refreshed with the media containing 5 or 25 mM glucose for another 2 h. ZBTB20 was visualized by Alexa 594 (Red), with the nuclei counterstained by DAPI (blue). Scale bars, 50 μm. The images were representative of three independent experiments. Data are represented as mean±s.e.m. *P<0.05; **P<0.01 versus indicated groups (Student's *t*-test).

Given the glucose responsiveness of ChREBP and its target genes, we then asked whether the expression and/or activity of Zbtb20 are regulated by dietary carbohydrates. To address this question, we first characterized Zbtb20 expression in adult male C57BL/6 mice under the fed and overnight fasted conditions. There was no significant difference between the two states in terms of Zbtb20 protein levels in the liver whole lysate (Fig. 6a). However, ChIP assay demonstrated that its binding to *ChREBP-α* promoter was significantly enhanced in the fed liver compared with the fasted counterpart (Fig. 6b), suggesting an increase in the activity. Of interest, western blot analysis revealed a robust elevation of Zbtb20 protein levels in the liver nuclear extracts from the fed mice compared with that from the fasted control, along with a mild but significant decrease in the cytoplasmic fraction (Fig. 6c). Consistently, immunohistochemical staining showed a marked increase in Zbtb20 signal intensity in the hepatic nuclei in fed condition compared with the fasted condition (Fig. 6d). Nevertheless, the nuclear localization of Zbtb20 was still detectable in fasted hepatocytes, the signal intensity of which was apparently weaker and highly heterogeneous. Given the unchanged total protein levels between the two states, we reasoned that the nuclear translocation and subsequent transcriptional activity of Zbtb20 may be nutritionally regulated in the liver.

Next, we further examined whether glucose could regulate the nuclear translocation of Zbtb20 in hepatocytes. After culture with 5 mM glucose for 36 h, the isolated primary hepatocytes displayed cytoplasmic localization of Zbtb20 by immunostaining. Then we refreshed the cells with the culture media containing different concentrations of glucose and cultured for another 2 h. Interestingly, the majority of hepatocytes exhibited the nuclear localization of Zbtb20 after the treatment of high (25 mM) rather than low (5 mM) glucose (Fig. 6e). These data suggested that glucose stimulation could regulate the nuclear translocation and subsequently the transcriptional activity of Zbtb20.

### Carbohydrate-induced glycolysis and lipogenesis require Zbtb20

**Figure 7:**
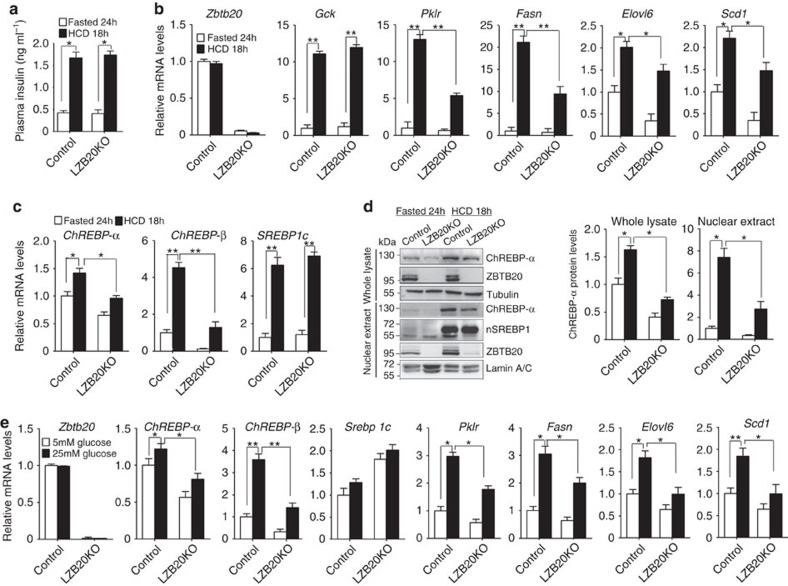
Zbtb20 is required for carbohydrate-induced activation of glycolytic and lipogenic genes. 3-4 month-old male control and LZB20KO mice were fasted for 24 h then or refed HCD for 18 h. *n*=5 per group. (**a**) Serum insulin levels were comparable between both genotypes. (**b**,**c**) mRNA expression levels of glycolytic and lipogenic genes (**b**) and transcriptional regulators (**c**). (**d**) Zbtb20 nuclear localization was increased after HCD feeding in control liver, and the activation and nuclear localization of ChREBP-α was impaired in LZB20KO liver, while SREBP1 was not affected. Lack of cytoplasmic protein contamination was confirmed in the nuclear extracts by probing tubulin (Supplementary Fig. 13). (**e**) Glucose-induced mRNA expression of glycolytic and lipogenic genes was decreased in LZB20KO hepatocytes *in vitro*. Primary hepatocytes from both genotypes were incubated under low glucose (5 mM) or high glucose (25 mM) conditions in the presence of 100 nM insulin for 6 h. *n*=5 per group. Error bars represent s.e.m. *P<0.05; **P<0.01 versus indicated groups (Student's *t*-test).

Next, we determined whether Zbtb20 is required for the induction of glycolytic and lipogenic genes by dietary carbohydrates. To address this question, we fasted control and mutant mice for 24 h, and then refed HCD for 18 h. HCD refeeding increased plasma insulin levels robustly and comparably in both control and mutant mice compared to the fasted control (Fig. 7a). In control liver, HCD refeeding led to a robust elevation in the mRNA levels of *Gck*, *Pklr*, *Fasn*, *SREBP1c* and *ChREBP-β*, and to some less extent, of *Scd1*, *Elovl6* and *ChREBP-α*, while Zbtb20 nuclear localization was substantially increased despite of its unchanged mRNA or total protein levels (Fig. 7b–d). Compared to control counterpart, HCD-refed mutant liver exhibited a remarkable decrease in the mRNA levels of *ChREBP-α*, *ChREBP-β*, *Pklr*, *Fasn*, *Elovl6* and *Scd1*, while the mRNA expression of *Srebp1c* and its target *Gck* was not affected. At the protein levels, ChREBP-α was increased by the comparable magnitudes in control and mutant liver whole lysate (1.6-fold in control versus 1.7-fold in mutant) or nuclear extracts (7.4-fold in control and 8.8-fold in mutant) after HCD refeeding compared to the fasted state. Notably, HCD-refed mutant mice exhibited a remarkable reduction in liver ChREBP-α protein levels in both the whole lysate (65.6%) and the nuclear fraction (60%) compared to control counterparts. Nevertheless, ChREBP-β protein was still not detected in the liver after HCD refeeding either in the whole lysate or the nuclear fraction, which is most likely because of its low expression levels. On the other hand, the protein levels of mature SREBP-1c were not significantly different between the two genotypes, consistent with their comparable plasma insulin levels and activation of its target gene *Gck*. These results indicate that Zbtb20 is indispensable for the efficient induction of a subset of glycolytic and lipogenic genes in the liver in response to dietary carbohydrates.

We then further examined whether Zbtb20 was required for glucose-activated ChREBP expression in primary hepatocytes *in vitro*. In the presence of insulin, treatment of wild-type hepatocytes with high glucose (25 mM) led to a slight and significant increase in *ChREBP-α* mRNA levels, and marked elevation in mRNA levels of *ChREBP-β*, *Pklr*, *Fasn*, *Elovl6* and *Scd1*. Remarkably, LZB20KO hepatocytes displayed an attenuated upregulation of mRNA levels of the above lipogenic genes in response to high glucose stimulation, while *SREBP1c* mRNA levels were significantly elevated compared to control (Fig. 7e). In addition, the insulin-stimulated AKT activation was not significantly different between control and mutant hepatocytes (Supplementary Fig. 9), indicating that the insulin signalling pathway was not impaired by the loss of Zbtb20. Taken together, these results suggest that Zbtb20 plays an important role in the full responsiveness of the hepatocytes to carbohydrate stimulation by activating a subset of glycolytic and lipogenic genes.

### The role of Zbtb20 in hepatic steatosis and insulin resistance

**Figure 8:**
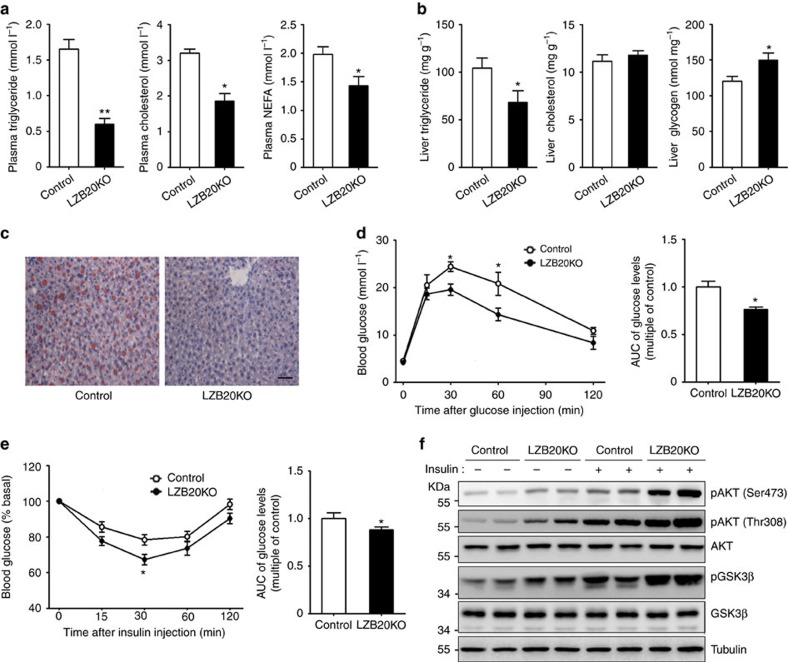
ZBTB20 deficiency protects against high-carbohydrate diet-induced hepatic steatosis and insulin resistance. Male control or LZB20KO mice at the age of 3–4 months were fed on high-carbohydrate diet (HCD) for 4 weeks. *n*=5 per group. (**a**) Plasma levels of TG, cholesterol and free fatty acid in the fasted state. (**b**) Liver contents of TG, cholesterol, and glycogen in the fed state. (**c**) Liver lipid was shown by Oil red O staining. Scale bar, 50 μm. (**d**) Glucose tolerance test was performed by intraperitoneal injection of 2 mg kg^−1^ of glucose, and displayed by area under curve. (**e**) Insulin tolerance test was performed by i.p. injection of insulin (0.75 U kg^−1^), and displayed by area under curve. (**f**) Phosphorylation of AKT and GSK3β in the liver was examined 3 min after insulin bolus via portal vein. Data are represented as mean±s.e.m. *P<0.05; **P<0.01 versus control (Student's *t*-test).

To explore the potential role of Zbtb20 in the pathophysiology of NAFLD, we first examined Zbtb20 expression in fatty liver. In ob/ob mice, which exhibit severe obesity and hepatic steatosis due to the leptin deficiency, Zbtb20 expression was substantially increased in the liver at both mRNA and protein levels compared to lean control (Supplementary Fig. 10a,b). However, in the fatty liver induced by chronic HCD feeding, Zbtb20 expression levels were similar as that from chow-fed normal control (Supplementary Fig. 10c–e). Then we further examined the effects of Zbtb20 deficiency on HCD-induced metabolic disorders. After 4 weeks of HCD feeding, there was no significant difference in body weight between control and mutant mice (control 25.68±0.58 g, mutant 26.40±0.54 g, *n*=8). Consistent with normal chow diet, HCD-fed mutant mice showed a slight and significant decrease in plasma glucose levels compared to control in fed state, whereas plasma insulin levels were comparable between the two genotypes despite a substantial increase relative to their chow-fed counterparts (Supplementary Fig. 11a,b). Compared to normal chow diet, HCD feeding resulted in a marked increase in plasma levels of triglyceride, cholesterol and free fatty acids in control rather than in mutant mice. Therefore, HCD-fed LZB20KO mice displayed even more prominent reductions in plasma levels of TG, cholesterol and free fatty acid compared to chow-fed counterparts (Fig. 8a). In the liver, HCD feeding led to a substantial elevation in the contents of TG and glycogen in both genotypes under the fed condition compared to their chow-fed counterparts. Remarkably, HCD-fed mutant liver exhibited a 30% decrease in TG contents and a 25% increase in glycogen contents compared to control group, while the liver cholesterol contents were still unchanged (Fig. 8b). Furthermore, Oil red O staining demonstrated a robust accumulation of lipid droplets on the liver sections from HCD-fed control mice, whereas LZB20KO liver displayed a substantial reduction in lipid droplets (Fig. 8c), indicating that deficiency of liver Zbtb20 could protect the mice from HCD-induced hepatic steatosis.

Considering that hepatic steatosis is closely associated with insulin resistance, we then evaluated the potential role of liver Zbtb20 in HCD-induced insulin resistance. As expected, HCD-fed LZB20KO mice displayed enhanced glucose disposal and improved insulin sensitivity relative to control counterparts (Fig. 8d,e). Moreover, the phosphorylation of AKT and GSK3β, which is indicative of the activation of insulin signalling pathway, was remarkably augmented in the liver from HCD-fed LZB20KO mice relative to control both under the basal fasted condition and after insulin bolus (Fig. 8f), while there was no significant difference in skeletal muscle or WAT between the two genotypes (Supplementary Fig. 11c). These results suggested that Zbtb20 deletion could improve HCD-induced liver insulin resistance.

Taken together, our identification of ZBTB20 as an essential regulator of hepatic lipogenesis provides insights into the regulation of lipid metabolism, which will help to unravel the biochemical basis of liver lipid synthesis, and facilitate the development of therapeutic strategy to treat hepatic steatosis and its related insulin resistance.

## Discussion

The present study establishes liver ZBTB20 as a key regulator of lipid homoeostasis. First, conditional ablation of hepatic Zbtb20 led to dysregulated lipid metabolism, which was mainly manifested by hypolipidemia with the reduction of plasma TG, TC and FFA. Second, the mice specifically lacking liver Zbtb20 displayed a substantial impairment in hepatic DNL and TG secretion, along with reduced liver TG accumulation. Third, Zbtb20 deficiency substantially impaired the expression of a subset of critical glycolytic and lipogenic genes in liver, including *Glut5*, *Pklr*, *Fasn*, *Elovl6* and *Scd1*, as well as two isoforms of *ChREBP*. These above genes exhibited a variable defect of activation in response to dietary carbohydrates in Zbtb20-deficient liver. Fourth, the nuclear translocation and transcriptional activity of Zbtb20 are regulated by glucose. Therefore, this work unravels a previously unrecognized role of Zbtb20 in lipid metabolism.

This study also provides insights into the hierarchy of transcriptional regulatory network of hepatic DNL. As the main lipogenic transcription factors, SREBP1c and ChREBP respond to the stimulation of insulin and carbohydrates, respectively, and synergistically regulate most of the glycolytic and lipogenic genes. Our results demonstrate that ZBTB20 governs hepatic lipogenesis largely through ChREBPs, with no significant effects on SREBP1c expression or activity. Both loss-of-function and gain-of-function data demonstrated that Zbtb20 regulated the expression of *ChREBP-α*, *ChREBP-β*, *Glut5*, *Pklr*, *Fasn*, *Elovl6* and *Scd1* in the liver. Among them, *ChREBP-β*, *Pklr*, *Fasn*, *Elovl6* and *Scd1* are well-established targets of ChREBPs^40^. Importantly, the regulation of these above five genes by Zbtb20 is most likely dependent of ChREBP, because inhibition of ChREBP protein expression by ChREBP-shRNA could largely abolish their activation by Zbtb20. Moreover, ChREBP-β overexpression could completely restore their expression in Zbtb20-deficient liver. On the other hand, the regulation of *Glut5* by Zbtb20 is most likely ChREBP-independent in the liver, with the evidence that Zbtb20 was capable of inducing *Glut5* expression even when ChREBP was inhibited. In addition, ChREBP-β overexpression did not significantly change liver *Glut5* expression in the presence or absence of Zbtb20. Although *Acc1* and *Gpat* are the important ChREBP targets for lipogenesis, their expression was not significantly changed by the loss of Zbtb20, probably because of their relatively weaker responsiveness to the decrease of ChREBP dosage than other targets (for example, *Elovl6*, *Scd1*) and the compensatory effects of other transcription factors (for example, SREBP1c). This is supported by the previous observation that *Acc1* is only mildly decreased in the ChREBP-null liver^8^, in which *Gpat* expression was not reported.

As to the potential regulatory mechanisms, our data support that Zbtb20 functions as a direct activator of *ChREBP-α* gene at the transcriptional levels, while its regulatory effect on *ChREBP-β* is most likely secondary to ChREBP-α. As a result, both two isoforms could activate the downstream targets involved in hepatic glycolysis and lipogenesis, such as *Pklr*, *Fasn*, *Elolv6* and *Scd1*. ChIP assay demonstrated the binding of Zbtb20 to *ChREBP-α* promoter but not that of *Pklr*, *Fasn*, *Elovl6* or *ChREBP-β*. In addition, reporter assay showed that Zbtb20 overexpression induced the transcriptional activity of *ChREBP-α* promoter but rather than that of *ChREBP-β*. Despite of its binding to *Scd1* gene in the ChIP assay, Zbtb20 had no effects on the transcriptional activity of *Scd1* promoter in the reporter assay. More importantly, the regulation of *Scd1* expression by Zbtb20 was also ChREBP-dependent. Therefore, the functional significance of the binding of Zbtb20 to *Scd1* gene remains unclear. On the other hand, our data did not support the possibility that Zbtb20 might regulate the transcriptional activity of ChREBPs through indirect mechanisms. First, the expression levels of Mlx, the obligate partner of ChREBP heterodimer, were not altered in the absence of Zbtb20. Second, the liver contents of G-6-P, which is involved in ChREBP activation^20^, were not affected by the loss of Zbtb20. Third, reporter assay showed that Zbtb20 overexpression had no effects on the transcriptional activity of ChREBP/Mlx. Put together, we postulate that Zbtb20 may translocate into nuclei in response to carbohydrate stimulation and thereby regulate the glycolytic and lipogenic enzyme genes at least partly through activating the expression of *ChREBP-α* and *ChREBP-β* via direct and indirect mechanisms, respectively (Supplementary Fig. 12).

Our previous studies have established ZBTB20 as a sequence-specific transcriptional repressor of the genes encoding alpha-fetoprotein (AFP) in liver^29^,^41^, fructose-1,6,-bisphosphotase 1 (FBP1) in pancreatic β cells^42^, IκBα in macrophages^43^ and Sox9 in hypertrophic chondrocytes^44^. Nevertheless, the identification of Zbtb20 as a transcriptional activator of *ChREBP-α* gene is also in agreement with our findings in other systems regarding to its transcriptional activity. For example, Zbtb20 binds to and promotes the expression of *Prl* gene encoding prolactin in pituitary, thereby driving the terminal differentiation of lactotrope^45^. In the suprachiasmatic nucleus (SCN) of the hypothalamus, Zbtb20 is required for the expression of *Prokr2* gene encoding prokineticin receptor-2, and modulates the outputs from the central circadian pacemaker^46^. However, in contrast to *Afp* and other target genes repressed by Zbtb20, we failed to identify the direct binding sequences of Zbtb20 in the promoters of *ChREBP-α*, *Prl* and *Prokr2* genes by conventional gel shift assay, which raised the possibility that Zbtb20 may exert its transcriptional activation as a cofactor by directly binding to transcriptional activators rather than DNA. Therefore, the transcriptional regulatory functions of ZBTB20 could be context-dependent, and the underlying mechanisms need further investigation.

This study also reveals a role of Zbtb20 in cholesterol metabolism. Zbtb20 deficiency leads to a decline of hepatic sterol synthesis, with no significant effects on the expression of the cholesterologenic transcription factor SREBP2 and its targets *Hmgcr* and *Hmgcs* at the mRNA or protein levels. It is possible that Zbtb20 may regulate the activity of HMGCR and/or HMGCS through unidentified posttranslational mechanisms, for example, protein modification or interaction. Another possibility is that Zbtb20 may regulate other rate-limiting enzymes beyond HMGCR and HMGCS in sterol synthesis. These raise an interesting subject about the role of Zbtb20 in sterol metabolism, which is under further investigation.

Lastly, this study demonstrates that hepatic ZBTB20 may play an important role in the pathophysiology of hepatic steatosis and insulin resistance. In contrast to the severe hypoglycemia observed in Zbtb20 global knockout mice^28^, deletion of liver Zbtb20 only causes a slight decrease in plasma glucose levels and a mild elevation in liver glycogen contents in the fed state, suggesting that liver Zbtb20 has limited effects on glucose homoeostasis under physiological condition. These results are consistent with our previous report that liver-specific overexpression of Zbtb20 can hardly rescue the severe hypoglycemia in its global knockout mice^28^. Therefore, we reason that extra-hepatic Zbtb20 is mainly responsible for glucose homoeostasis, which we are currently exploring using various conditional knockout mice. Noteworthy, a recent study reported that sporadic point mutations of ZBTB20 in the zinc finger domains are associated with Primrose syndrome^47^, a condition characterized by increased growth of the brain and taller stature, hypotonia, intellectual disability and autism. Interestingly, individuals with Primrose syndrome develop diabetes in adulthood, which is consistent with the role of Zbtb20 in pancreatic β cells regulating glucose metabolism and insulin secretion^42^. In the present study, we observed that specific deletion of liver Zbtb20 improved HCD-induced hepatic steatosis and insulin resistance. Biochemical analysis demonstrated that the insulin sensitivity was enhanced in the liver rather than skeletal muscle and white adipose tissue from Zbtb20-deficient mice. Given the close association between hepatic steatosis and insulin resistance^1^, it is likely that the amelioration of insulin resistance may be caused by the improved hepatic steatosis in the absence of Zbtb20. Of note, our data are consistent with the observations that inhibition of ChREBP expression or activity improves hepatic steatosis and insulin resistance in mice^9^,^48^. Regarding to its potential role in the pathophysiology of hepatic steatosis, unexpectedly, Zbtb20 expression was not significantly altered in HCD-induced fatty liver at the mRNA or protein levels. One possibility is that the activity of liver Zbtb20 may be enhanced in the pathogenesis of hepatic steatosis. It is interesting to note its substantial increase in the liver from ob/ob mice, the potential role of which in the hepatic steatosis and insulin resistance is under investigation. Taken together, the significance and mechanism of Zbtb20 in glucose metabolism is rather complex and may be tissue-dependent.

Collectively, our identification of ZBTB20 as a critical regulator of ChREBP provides novel insight into the hierarchy of transcriptional regulatory network of hepatic lipogenesis, which will help to unravel the biochemical basis of NAFLD and develop a therapeutic approach to this disease.

## Methods

### Animals

We generated liver-specific ZBTB20 knockout mice (LZB20KO) by crossing *Zbtb20*^*flox*^ mice with *Alb-Cre* transgenic mice as previously described^29^, and have crossed the heterozygote *Zbtb20*^*f/+*^; *Alb-Cre* onto C57BL6/J background for at least eight times before intercrossing to homozygosity.

### Animal experiments

All animal experiments were performed in accordance with the approval obtained from the Second Military Medical University Animal Ethics Committee. Mice were housed in a specific pathogen-free barrier facility under controlled temperature and light, with free access to water and standard chow diet unless otherwise specified. In some experiments, we fed the mice with a high-carbohydrate diet (HCD, 72.2% carbohydrate, 1% fat and 26.8% protein in calorie). We used sex-matched littermates for all the experiments, and sacrifice mice after overnight 16-h fasted or in the early light phase under fed condition unless otherwise noted. We isolated tissues immediately, weighed and kept at −80 °C after snap freezing in liquid nitrogen. We measured fat and lean mass by dual-energy X-ray absorptiometry using a PIXImus mouse densitometer (GE Healthcare). We performed indirect calorimetry using the Oxymax Comprehensive Lab Animal Monitoring System (Columbus Instruments), and collected data for 3 days after 2 days of acclimation in metabolic chamber^42^.

### Metabolic assays

We measured blood glucose using a glucose monitor (One Touch Ultra, Lifescan, Johnson & Johnson, Milpitas, California); plasma insulin with an ultrasensitive mouse insulin ELISA kit (Mercodia, Sweden); plasma and liver TG, cholesterol, and NEFA by colorimetric assays (Sigma and Wako). We extracted TG and cholesterol from liver with acetone for colorimetric assays. We extracted and digested hepatic glycogen with amyloglucosidase, and measured with a glucose oxidase quantitation assay (Sigma)^28^. For lipoprotein separation, we pooled serum samples of five mice per genotype, resolved by fast performance liquid chromatography (FPLC) on a gel filtration column Superose 6 (Bio-Rad), and collected elutes in 0.5 ml fractions at a flow rate of 0.7 ml min^−1^. For VLDL secretion assays, we fasted mice for 4 hours before i.p. injection of 0.5 mg kg^−1^ tyloxapol (Sigma), and performed tail bleedings at indicated time points for plasma TG measurements. For glucose tolerance test, adult mice were fasted for 16 h prior to i.p. injection of D-glucose (2 g kg^−1^ body weight). For insulin tolerance testing, mice in the randomly fed state were i.p. injected with 0.75 U kg^−1^ body weight of human regular insulin (Sigma). We collected blood before injection and at indicated time points after injection to measure glucose and insulin levels. For plasma lipid clearance test, we fed the animals by gavage with a bolus dose of olive oil (15 μl g^−1^ body weight) after a 6-h fasted period, and collected blood samples from the tail vein immediately before and at 1, 2 and 3 h after the oil bolus. For insulin sensitivity analysis, the mice under the fasted condition were subjected to insulin bolus injection (10 U kg^−1^) via portal vein, and the liver, skeletal muscle and white adipose tissues were collected for biochemical analysis at 3, 5 and 7 min after bolus, respectively.

### Histology and Oil red O staining

Pieces of the liver fixed in 4% paraformaldehyde were embedded in paraffin and stained with hematoxylin and eosin. For Oil Red O staining, we froze liver tissues in OCT compounds before cutting at 8 μm, and performed staining according to standard protocol. Immunohistochemical analysis of Zbtb20 was performed on liver sections by overnight incubation at 4 °C with home-made mouse anti-ZBTB20 monoclonal antibody 9A10 (1:1,000), and subsequent visualization with Alexa Fluor 594 and nuclear counterstaining with DAPI^49^.

### *In vivo* lipogenesis assay

We measured hepatic DNL *in vivo* as using [^3^H]-labelled water. Briefly, mice were i.p. injected with 0.5 ml of 0.15 N NaCl containing 0.2 mCi of [^3^H]-water/100 g body weight. One hour later, we excised liver samples (∼700 mg per animal), heated them at 90 °C for 5 h in a mixture of 1.5 ml of 4 M KOH and 1.5 ml of 95% ethanol, and then mixed with 4 ml of hexane. After centrifugation, we collected the organic phase, and dried for [^3^H]-sterol assay. The aqueous phase (3 ml) was acidified with 0.75 ml of 10 M H_2_SO_4_ before mixing with 4 ml hexane, and subjected to centrifugation. Then we washed the organic phase with 3 ml of distilled water, and dried for the determination of [^3^H]-labelled fatty acids.

### Primary cultures of hepatocytes

Mouse primary hepatocytes were isolated by collagenase Ι (CLS 1, Worthington) perfusion. Hepatocytes were resuspended in M199 media supplemented with 10% fetal bovine serum, 100 nM insulin, and 100 nM dexamethasone in the presence of 5.5 mM glucose, plated at a density of 1.5 × 10^6^ cells per 35 mm dish and allowed to attach for 4 h. For Zbtb20 nuclear translocation experiments, wild-type hepatocytes were incubated for 36 h in the M199 medium with 5 mM glucose, then refreshed with the medium containing 5 or 25 mM glucose for 2 h before being subjected to immunohistochemical staining. For glucose stimulation experiments, hepatocytes from wild-type and LZB20KO mice were incubated for 24 h in the M199 medium with 5 mM glucose, then refreshed with the medium containing 5 or 25 mM glucose for 6 h before harvest for mRNA and protein expression analysis.

### Adenoviral gene transduction into hepatocytes *in vivo*

Replication-incompetent recombinant adenovirus expressing ChREBP-shRNA was generated using BLOCK-iT Adenoviral RNAi expression system (Invitrogen) according to the manufacturer's instructions. Briefly, we selected a shRNA against mouse ChREBP (NM_021455) at +859/877 with the sequence of 5′-TGTTGGCAATGCTGACATG-3′, which has been validated to inhibit ChREBP expression^9^. These two DNA oligonucleotides, 5′-caccGTGTTGGCAATGCTGACATGcgaaCATGTCAGCATTGCCAACA-3′ (forward) and 5′-aaaaTGTTGGCAATGCTGACATGttcgCATGTCAGCATTGCCAACAC-3′ (reverse), were synthesized and annealed to generate dsDNA, which was subsequently cloned into the pENTR/U6 vector under the control of human U6 promoter. The U6-shRNA cassette was transferred to the adenoviral expression plasmid by recombination reaction. Then the isolated adenoviral expression clones were digested with PacI to expose the inverted terminal repeats and transfected into 293A cells using Effectene (Qiagen) for production of a crude adenoviral stock. An adenovirus expressing β-galactosidase shRNA (Ad-LacZ-shRNA) was constructed as a control shRNA vector. Recombinant adenoviral vector expressing human ZBTB20 or mouse ChREBP-β was constructed by inserting the corresponding cDNA into the adenoviral vector pAd-Track (Stratagene) under the control of CMV promoter, and *in vitro* recombination with adenoviral backbone vector pAdEasy-1 and subsequent viral packaging in 293A cells were performed according to the instructions of the manufacturer. The recombinant adenoviruses were amplified at large scale, purified by CsCl ultracentrifugation, and subjected to dialysis against PBS before titration in 293A cells by the standard plaque-forming assay as described previously^45^. Adenovirus expressing GFP was also prepared as control. To investigate Zbtb20-regulated gene expression, adult male LZB20KO mice were injected via tail vein with Ad-shChREBP or control Ad-shLacZ adenoviruses (0.3 OD per mouse) together with Ad-ZBTB20 or Ad-GFP as control (0.2 OD per mouse). Five days later, the liver was harvested under the 4 h fasted condition for gene expression analysis. To evaluate the rescuing effects of ChREBP, adult male control or LZB20KO mice were injected through tail vein with ChREBP-β-expressing adenoviruses (Ad-ChREBP) or Ad-GFP as control (0.2 OD), and gene expression and phenotypes were analysed 1 week later.

### mRNA expression analysis

We extracted total RNA from primary hepatocytes or tissue samples (∼50 mg) with TRIzol (Invitrogen) homogenates or RNeasy kit (Qiagen GmbH, Hilden). cDNA was synthesized with a ReverTra Ace-a Kit (Toyobo) from total RNA after DNase I treatment, and real-time PCR was performed with a QuantiFast SYBR Green PCR Kit (Qiagen) with included *36B4* gene as internal control in every plate. The PCR specific primers for each gene are listed in Supplementary Table 3.

### Protein expression analysis

Whole lysate was extracted with a buffer containing 50 mM Tris-HCl (pH 7.4), 150 mM NaCl, 1 mM EDTA, 1 mM Na_3_VO_4_, 1 mM NaF, 1% Nonidet P-40, 0.25% Na-deoxycholate, 1 mM PMSF, 1 μg ml^−1^ leupeptin, 1 μg ml^−1^ aprotinin and 1 μg ml^−1^ pepstatin. Nuclear and cytoplasmic extracts were prepared using the NE-PER nuclear and cytoplasmic extraction reagent kit (Pierce Biotechnology) according to the manufacturer's instructions. Proteins were subjected to SDS-PAGE analysis on a 10% gel, transferred to polyvinylidene difluoride (PVDF) membranes (PolyScreen), and incubated with the appropriate primary antibody by standard protocols. After probed with HRP-conjugated secondary antibodies, immunoreactive proteins were visualized with ECL Western blotting detection reagents (Pierce Biotechnology, Rockford). Monoclonal mouse tubulin and Lamin A/C (Proteintech) antibodies were used as loading controls. The nuclear extracts were also reprobed to check the lack of tubulin as quality control. The antibody information and working dilutions were listed in Supplementary Table 4. Uncropped scans of the most important western blots were shown in Supplementary Fig. 13.

### ChIP analysis

Liver (1 g) was crosslinked with 1% formaldehyde for 7 min at room temperature before stop by adding 0.5 M glycine. After sonication, fragmented chromatin was incubated overnight at 4 °C with anti-ZBTB20 antibody 9A10 (home-made, 1 μg per reaction), anti-acetylated histone 3 (Millipore, Cat #17-10050, 0.5 μg per reaction) as positive control or isotype IgG (Upstate, 1 μg per reaction) as negative control, and followed by incubation with Dynabead-conjugated Protein G (Invitrogen). Purified chromatin DNA was subjected to real-time PCR analysis with the primers for gene promoter, the sequence of which is were listed in Supplementary Table 5. The data were analysed using the formula of 2^−ΔΔCt^, where ΔΔCT=(Ct[IP]−Ct[input])_SA_−(Ct[IP]–Ct[input])_NS_. SA=specific antibody, NS=non-specific antibody. Three independent ChIPs were performed.

### Reporter assay

Human ZBTB20 expression plasmid made in the lab, mouse ChREBP-α promoter (−3.8 kb, a gift from Dr Koshi Hashimoto) and the reporter containing ChoER in the promoter (a gift from Dr Catherine Postic) have been previously described^7^,^22^,^29^, respectively. Serial truncated promoters of mouse ChREBP-α were made by restrictive deletion. The mouse ChREBP-β (−0.7 kb) and Scd1 promoters (−1.4 kb) were cloned by PCR using genomic DNA as template and inserted into pGL4-basic vector. Mouse Mlxβ cDNA was cloned by RT-PCR from the liver, and ChREBP-β cDNA cloned by PCR using ChREBP-α-expressing plasmid (a gift from Dr Catherine Postic) as template, both of which were introduced into the expression vector pCMV-tag2 (Stratagene) to express with a FLAG-tag at the N terminal, respectively. All the plasmids were confirmed by DNA sequencing. Zbtb20-deficient primary hepatocytes or human embryonic kidney cells 293 T (ATCC) were transfected with plasmids using Lipofectamine 2000, and the luciferase activity was detected 48 h after transfection using Dual-luciferase assay kit (Promgea) and normalized by the internal control RL-TK.

### Statistical analysis

All values unless otherwise indicated are expressed as mean±s.e.m. Statistical analyses were carried out using Student's *t* test or ANOVA followed by *post-hoc* comparisons, and the null hypothesis was rejected at the 0.05 level.

### Data availability

All relevant data are available upon reasonable request from the authors.

## Additional information

**How to cite this article:** Liu, G. *et al*. Regulation of hepatic lipogenesis by the zinc finger protein Zbtb20. *Nat. Commun.* **8,** 14824 doi: 10.1038/ncomms14824 (2017).

**Publisher's note:** Springer Nature remains neutral with regard to jurisdictional claims in published maps and institutional affiliations.

## Supplementary Material

Supplementary Information Supplementary figures and supplementary tables.
