# Supplementary material for: Regulation of hepatic lipogenesis by the zinc finger protein Zbtb20
Source: Nat Commun. 2017 Mar 22;8:14824. doi: 10.1038/ncomms14824 (PMC5364431; doi:10.1038/ncomms14824)
Supplement: Supplementary Information — Supplementary figures and supplementary tables. [file ncomms14824-s1.pdf]

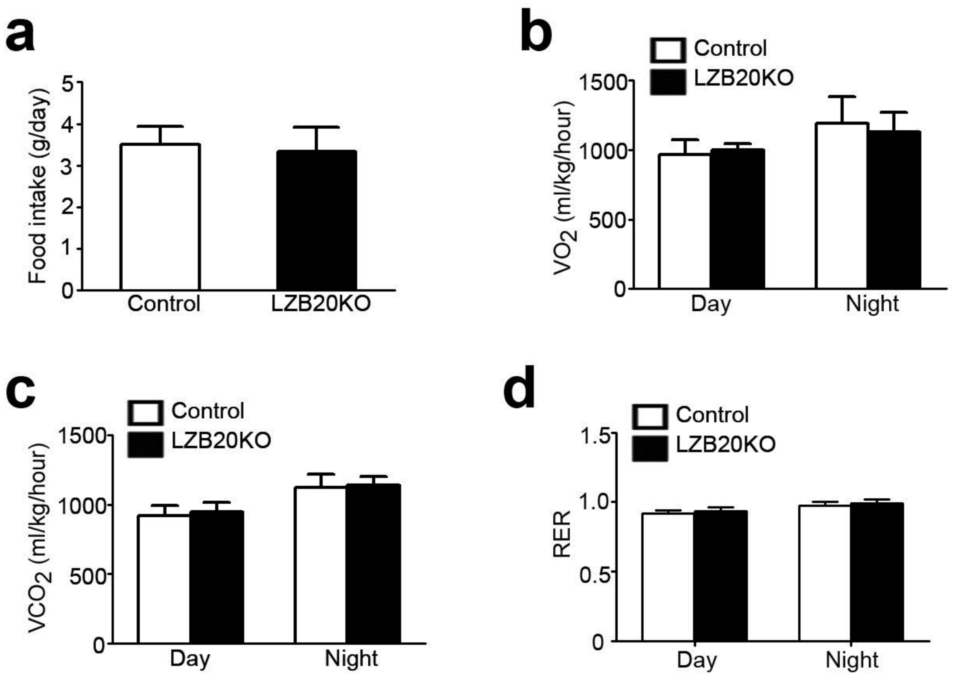

Supplementary Figure 1. LZB20KO mice have normal food intake and energy expenditure. The 3～4 month-old male control or liver-specific ZBTB20 knockout mice on normal chow were measured for food intake (a), and their energy expenditure was determined by an open circuit indirect calorimeter (b-d). Mice were acclimated in the monitoring chambers for 2 days followed by data collection for 3 days. The rates of O_2 consumption (b) and CO_2 production (c), and the respiratory exchange ratio (RER, VCO_2/VO_2) (d) were measured. n=7 per group. Data are presented as the means ± SEM, with no significant difference between both groups (Student's t-test).

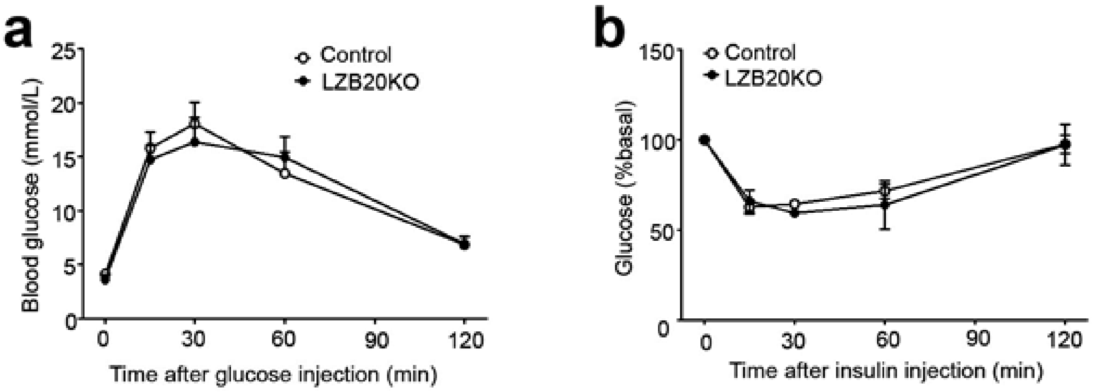

Supplementary Figure 2. LZB20KO mice on normal chow exhibited normal glucose tolerance and insulin sensitivity. The 3～4 month-old male control or liver-specific ZBTB20 knockout mice were on normal chow. **(a-b)** Glucose tolerance test (a) and insulin tolerance test (b) were performed by intraperitoneal injection of 2 mg/kg of glucose or 0.75 U/kg of insulin, respectively. n=5 per group. Data are represented as mean±SEM, with no significant difference between both groups (Student’s t-test).

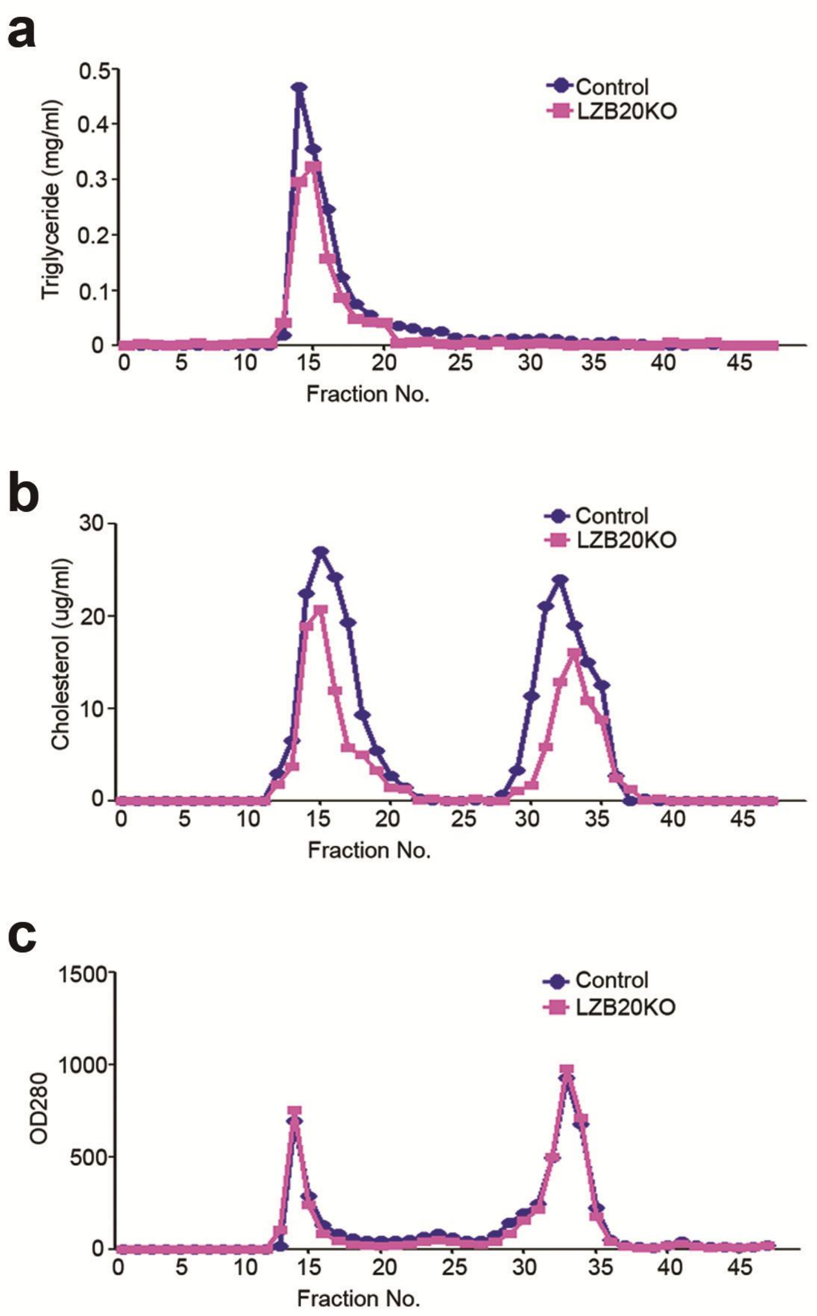

Supplementary Figure 3. FPLC analysis of plasma lipoprotein particles from tyloxapol-injected mice. Male mice on normal chow were fasted overnight and injected with tyloxapol（500mg/kg body weight）3 hours prior to sacrifice to enrich plasma VLDL. Plasma samples were pooled from 6 mice per group for FPLC separation of lipoprotein particles. Distribution of TG **(a),** cholesterol **(b),** and total protein **(c)** was presented. The plots were representative of 3 independent experiments.

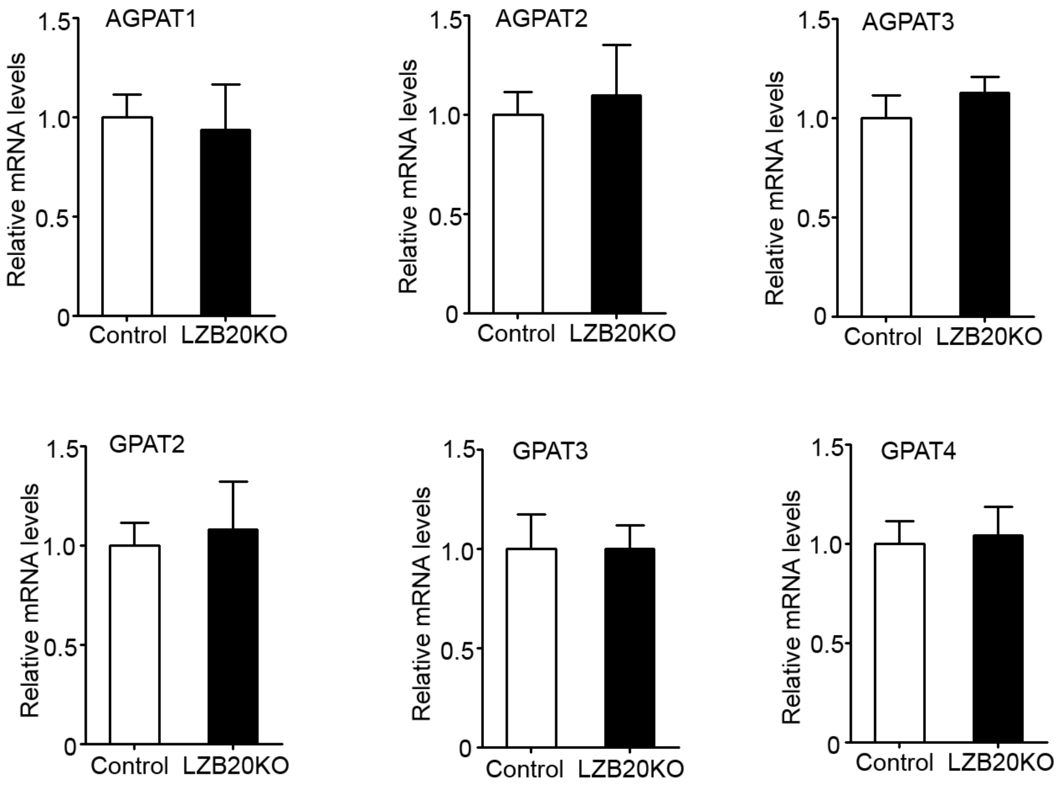

Supplementary Figure 4. mRNA expression levels of the genes involved in triglyceride synthesis in the liver of LZB20KO mice. Total RNA was extracted from the livers of male control or LZB20KO mice on normal chow, and mRNA levels of the indicated genes were determined by quantitative RT-PCR with *36B4* gene as internal control. n=6 per group. Data are represented as mean±SEM, with no significant difference between both groups (Student's t-test).

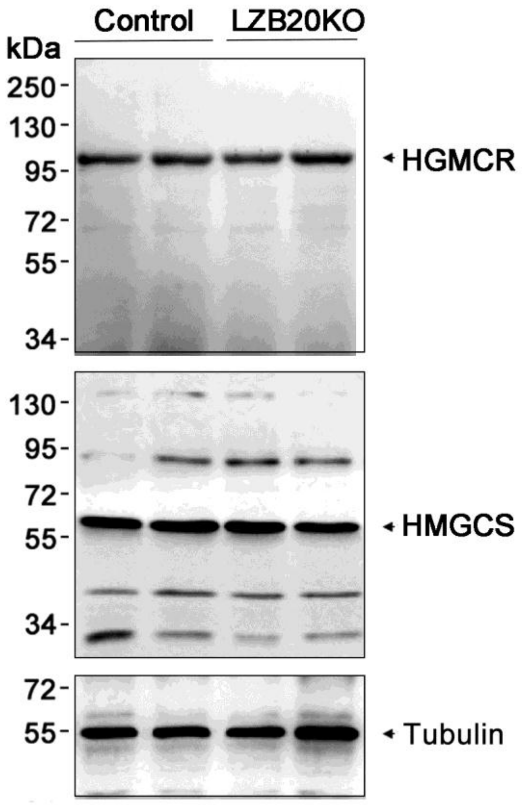

Supplementary Figure 5. Normal expression of HMGCR and HMGCS proteins in LZB20KO liver. Western blot analysis for HMGCR and HMGCS in the liver from male normal chow-fed control and LZB20KO mice under the fasted condition. The blots were representative of 3 independent experiments.

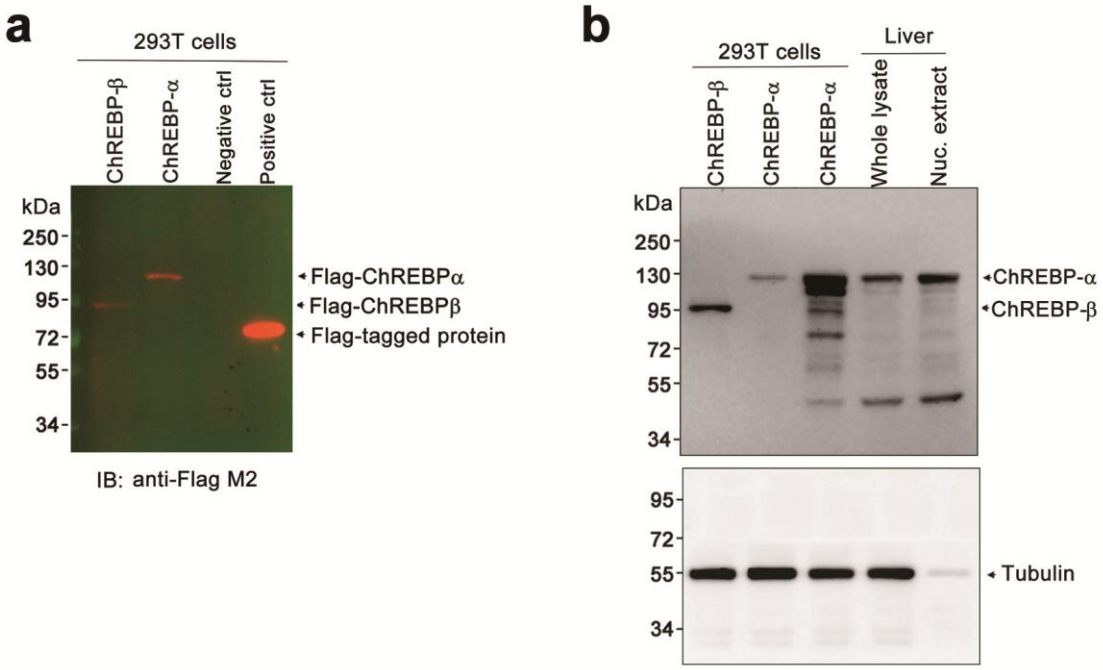

Supplementary Figure 6. ChREBP-α is the predominant form of ChREBP protein in normal liver. (a-b) Flag-tagged ChREBP-α and ChREBP-β proteins were specifically detected from their overexpressed 293T cells by the anti-FLAG M2 monoclonal antibody (a) and anti-ChREBP polyclonal antibodies (b), respectively. Endogenous liver ChREBP was also detected using anti-ChREBP antibodies. 293T cells were transfected with the different amounts of plasmids expressing Flag-tagged ChREBP-α or ChREBP-β, or negative and positive control (ctrl), and then the whole lystate was prepared 48h post-transfection. The whole lysate and nuclear (nuc.) extract were also prepared from normal liver in fed state. The anti-ChREBP antibodies recognized both isoforms of ChREBP overexpressed in hepatocytes. Only endogenous ChREBP-α was detected in the liver, while endogenous ChREBP-β was almost undetected likely due to its physiologically low expression. The blots were representative of two independent experiments.

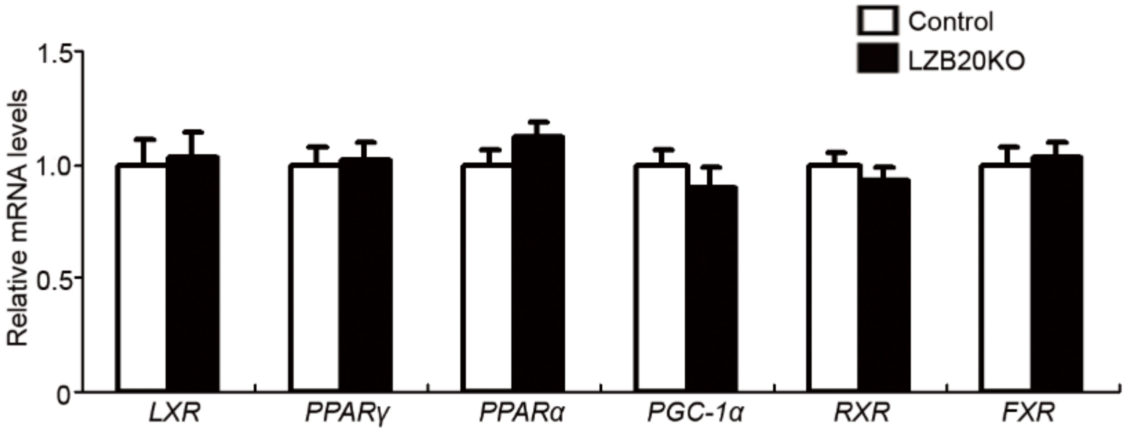

Supplementary Figure 7. mRNA levels of the transcription factors involved in lipid metabolism in LZB20KO liver. Total RNA was extracted from the livers of control or LZB20KO mice on normal chow, and mRNA levels of the indicated genes were determined by quantitative RT-PCR with *36B4* gene as internal control, which showed no significant difference between both groups. n=6 per group. Data are represented as mean$\pm$SEM. $p>0.05$ vs control (Student's t-test).

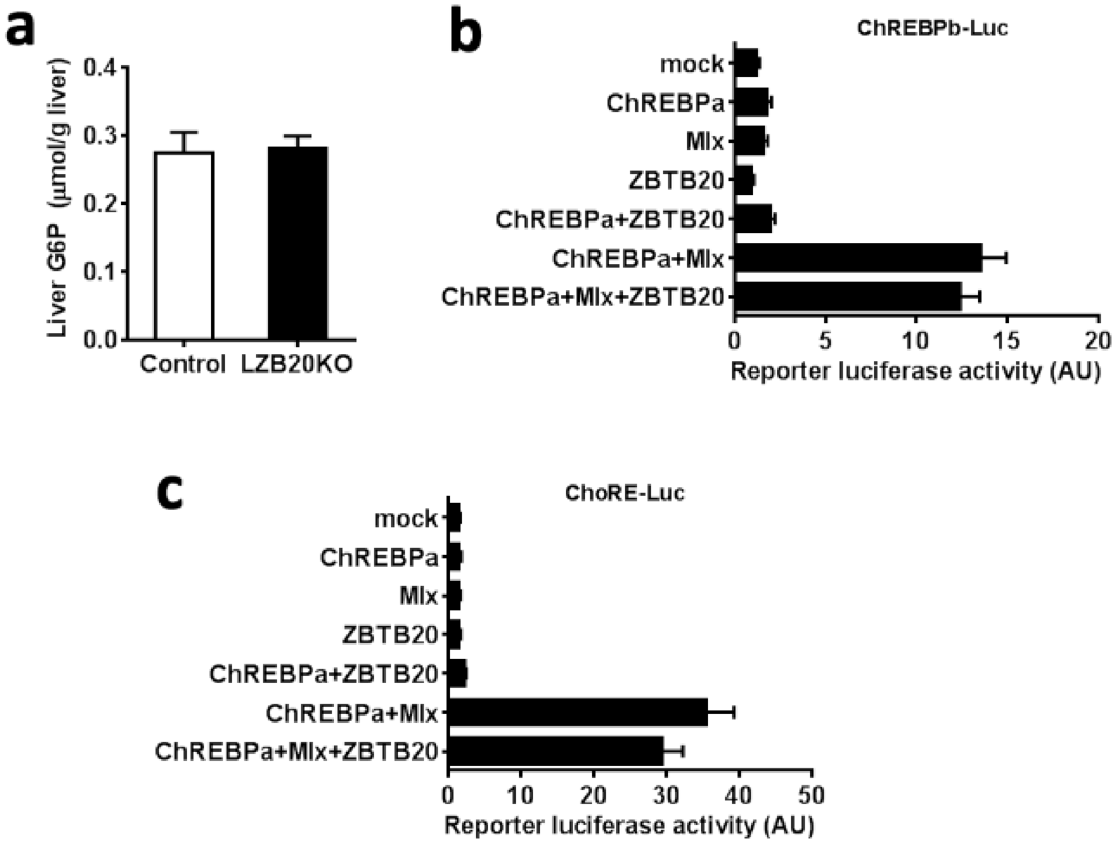

Supplementary Figure 8. ZBTB20 has no effects on the transcriptional activity of ChREBP proteins. (**a**) Liver glucose-6-phosphate (G6P) contents were not different between chow-fed control and LZB20KO mice under fed condition. n=5 per group. (**b-c**) Luciferase assay in 293T cells showed that ZBTB20 did not significantly affect the transcriptional activity of *ChREBPb* promoter or the ChoRE reporter. These two reporters were robustly active in 293T cells in the presence of ChREBP-α/Mlx, the activity of which was normalized by renilla activity as internal control. Data are represented as mean± SEM. There were no significant difference between control and LZB20KO groups (a) or between ChREBPa+Mlx and ChREBPa+Mlx+ZBTB20 groups (b and c) according to Students't-test.

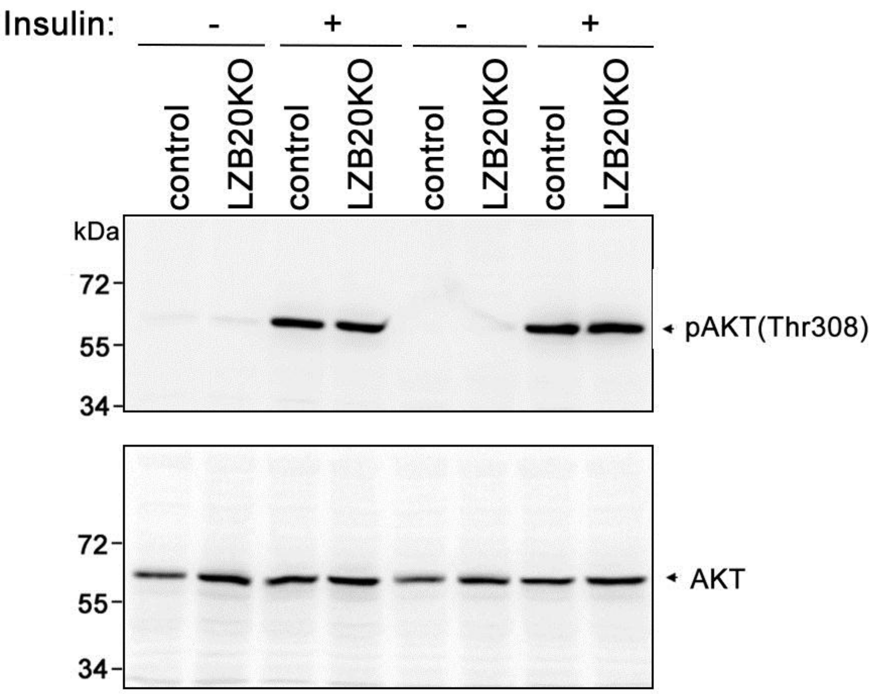

Supplementary Figure 9. Insulin signaling pathway is not altered in primary hepatocytes from normal chow-fed mice. Primary hepatocytes were isolated from male control or LZB20KO mice on normal chow, and plated at a density of 6 x 10^5 cells per well in six-well plates. After adherence of 4 hr, the hepatocytes were washed twice with PBS and cultured in serum-free M199 medium with 5.5 mM glucose for 2 hours, followed by treatment with or without 100 nM insulin for 10 min. Cell lysates were subjected to Western analyses for phospho-Akt (Thr^{308}), and the blots were stripped before reprobing with anti-Akt antibodies. The blots were representative of three independent experiments.

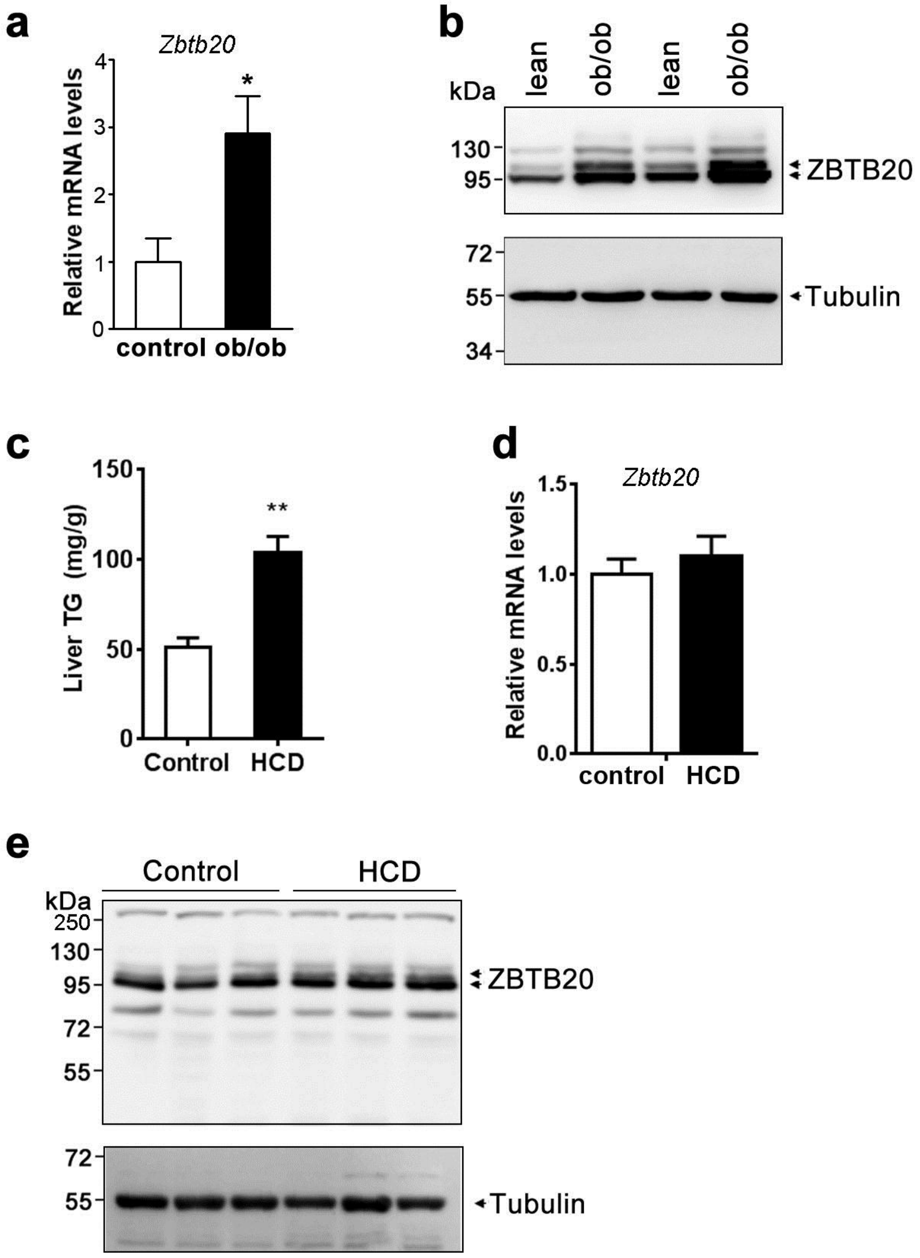

Supplementary Figure 10. ZBTB20 expression is increased in the fatty liver from ob/ob mice. (**a-b**) The adult male ob/ob and control lean mice were on normal chow. ZBTB20 expression was substantially increased in the liver at the mRNA levels by qRT-PCR analysis (a) and protein levels in the whole lysate (b) compared to lean control. (**c-e**) Male adult C57BL/6 mice developed fatty liver after HCD feeding for 4 weeks (**c**), and ZBTB20 expression was not changed at the mRNA levels (**d**) or total protein levels (**e**) compared with normal chow-fed control. Data are presented as the means ± SEM. n=6 per group. * p<0.05, **p<0.01 versus control (Student's t-test). The blots were representative of three independent experiments.

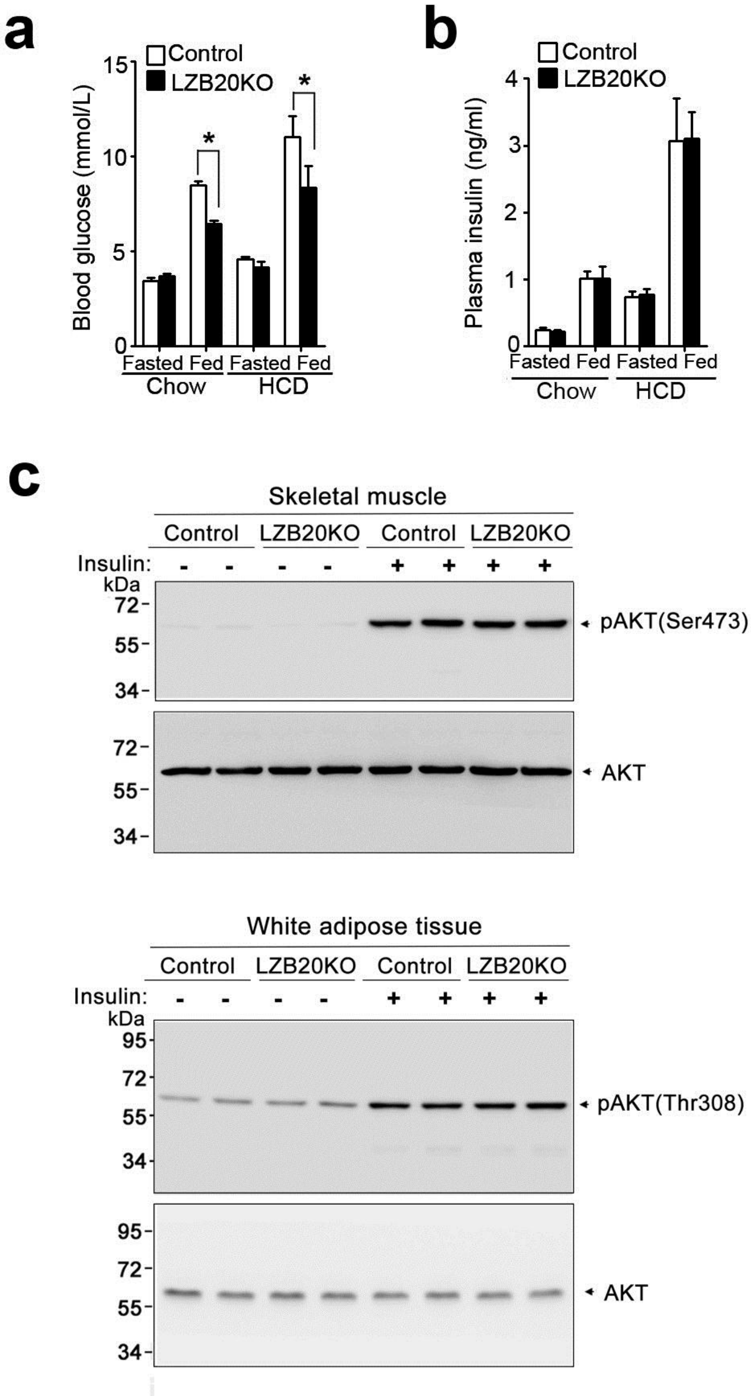

Supplementary Figure 11. Glucose homeostasis and extrahepatic insulin signaling in HCD-fed LZB20KO mice. Control and mutant male mice were fed HCD for 2 months. (**a-b**) Blood glucose (a) and plasma insulin (b) levels in fasted condition. n=7 per group. (**c**) Activation of AKT by Insulin was not altered in skeletal muscle and WAT in HCD-fed mice by the loss of liver ZBTB20. Mice were fasted overnight before insulin bolus via portal vein, and skeletal muscle and WAT were harvested at 5 and 7 min post insulin bolus, respectively. The blots were representative of three independent experiments. Data are presented as the means ± SEM. *, $p<0.05$ vs indicated control (Student's t-test).

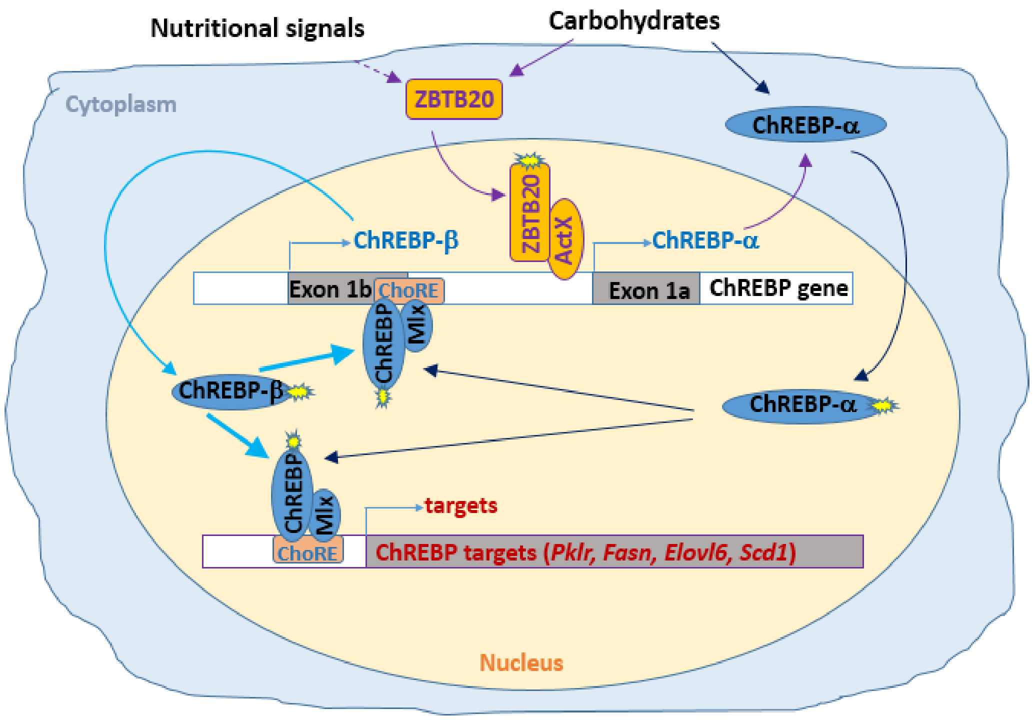

Supplementary Figure 12. Schematic illustration for the regulation of glycolysis and lipogenesis by ZBTB20. In response to nutritional signals and carbohydrate stimulation, ZBTB20 undergoes nuclear translocation from hepatic cytoplasm, and subsequently induces ChREBP-α gene expression, which probably involves the interaction with an unknown transcriptional activator (ActX). As a result of carbohydrate stimulation, the activated ChREBP-α protein enters into nuclei from cytoplasm, thereby inducing ChREBP-β transcription via the ChoER element partially located in exon 1b. Both isoforms of ChREBP protein activate the glycolytic and lipogenic enzyme genes (*Pklr, Acc1, Fas, Elovl6, Scd1,* etc) via the ChoER elements in their promoters in the form of ChREBP/Mlx heterodimer, while ChREBP-β is more potent than ChREBP-α in the transcriptional activity. ChREBP-β can also activate itself in a positive feed-back manner.

Fig.3f

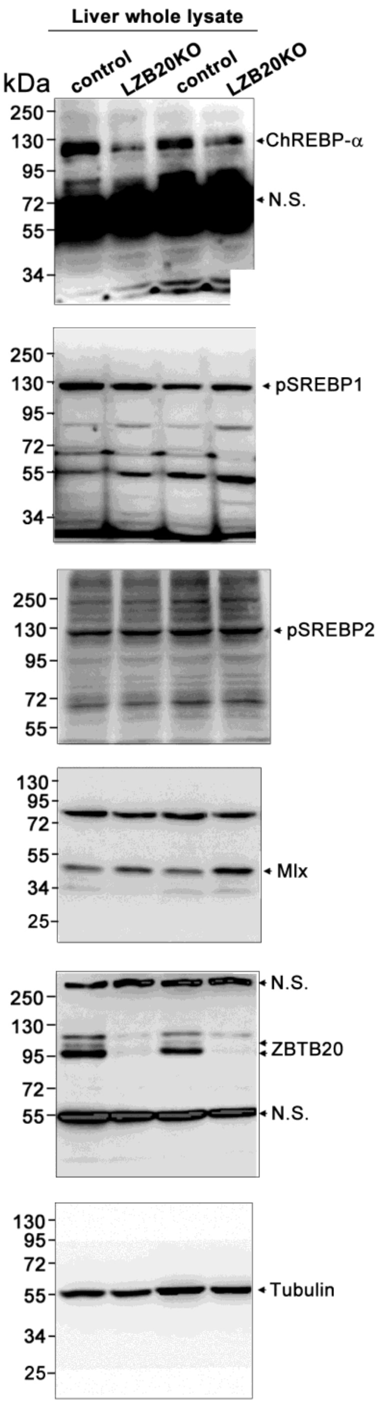
Liver whole lysate
kDa
control
LZB20KO
control
LZB20KO
250
130
95
72
55
34
ChREBP-α
N.S.
pSREBP1
pSREBP2
Mlx
N.S.
ZBTB20
N.S.
Tubulin
25

Fig.3g

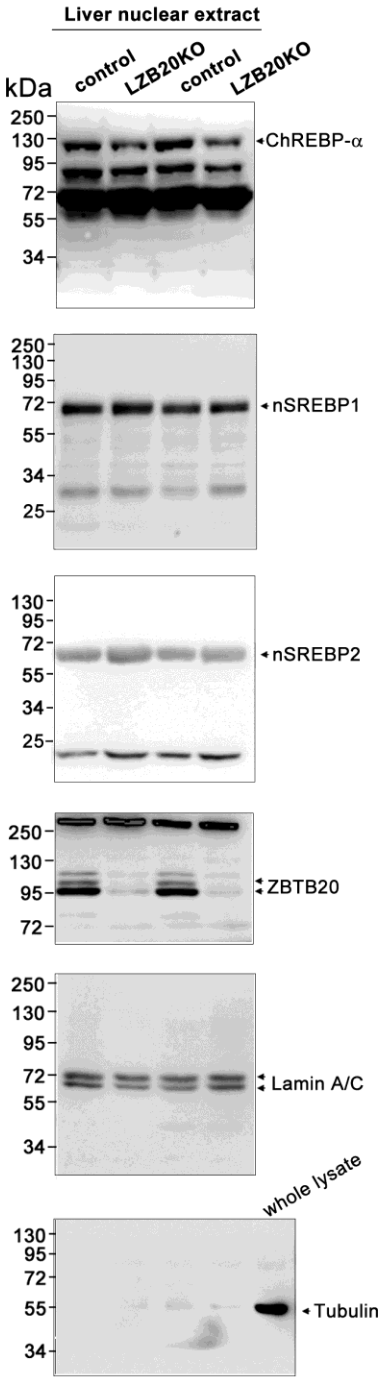
Liver nuclear extract
kDa
control
LZB20KO
control
LZB20KO
250
130
95
72
55
34
25
ChREBP-α
nSREBP1
nSREBP2
ZBTB20
Lamin A/C
whole lysate
Tubulin

Fig.4c

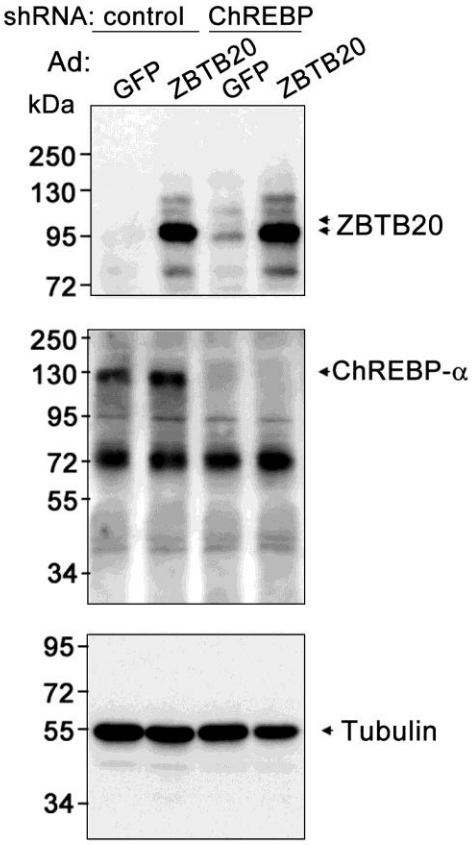

shRNA: control ChREBP
Ad: GFP ZBTB20 GFP ZBTB20
kDa
250
130
95
72
ZBTB20
250
130
95
72
55
34
ChREBP-α
95
72
55
34
Tubulin

Fig.5b

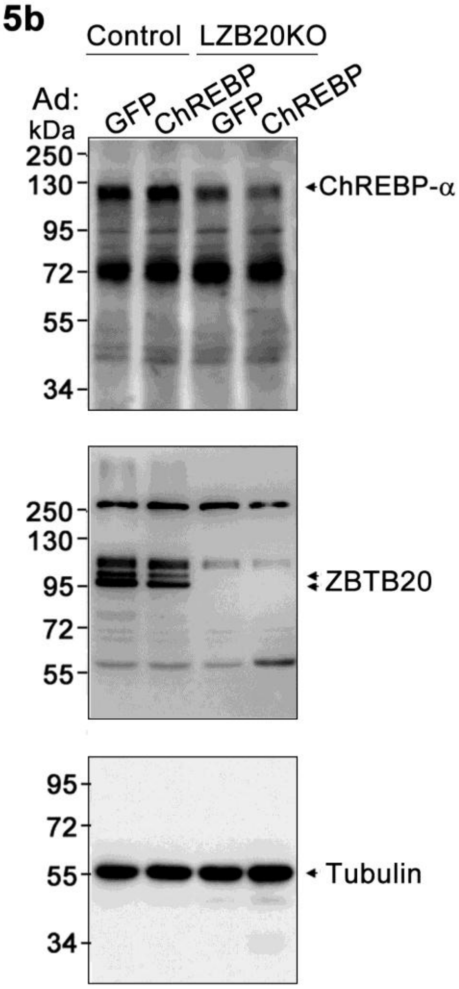

Control LZB20KO
Ad: GFP ChREBP GFP ChREBP
kDa
250
130
95
72
55
34
ChREBP-α
250
130
95
72
55
ZBTB20
95
72
55
34
Tubulin

Fig.6a

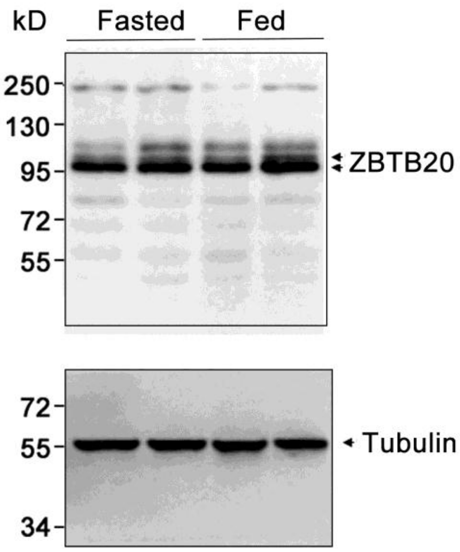

kD
Fasted
Fed
250
130
95
72
55
ZBTB20
72
55
34
Tubulin

Fig.6c

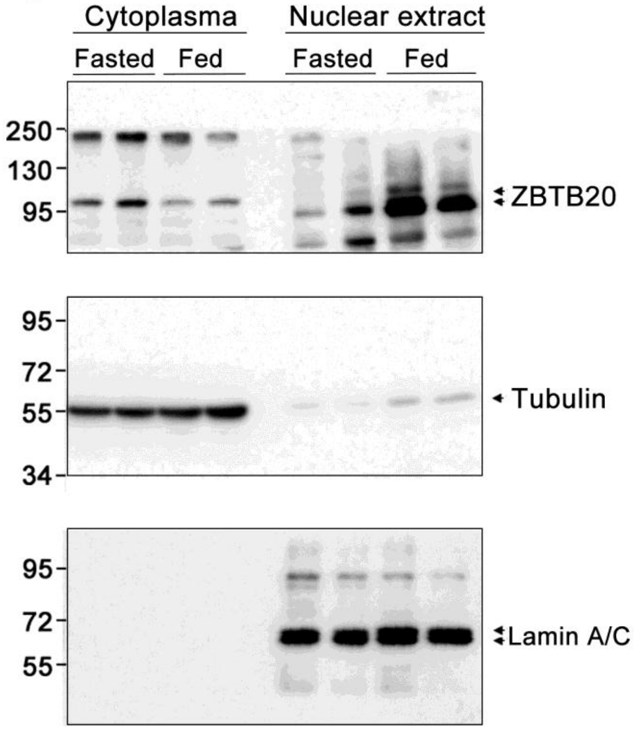

Cytoplasma
Nuclear extract
Fasted
Fed
Fasted
Fed
250
130
95
ZBTB20
95
72
55
34
Tubulin
95
72
55
Lamin A/C

Fig.7d

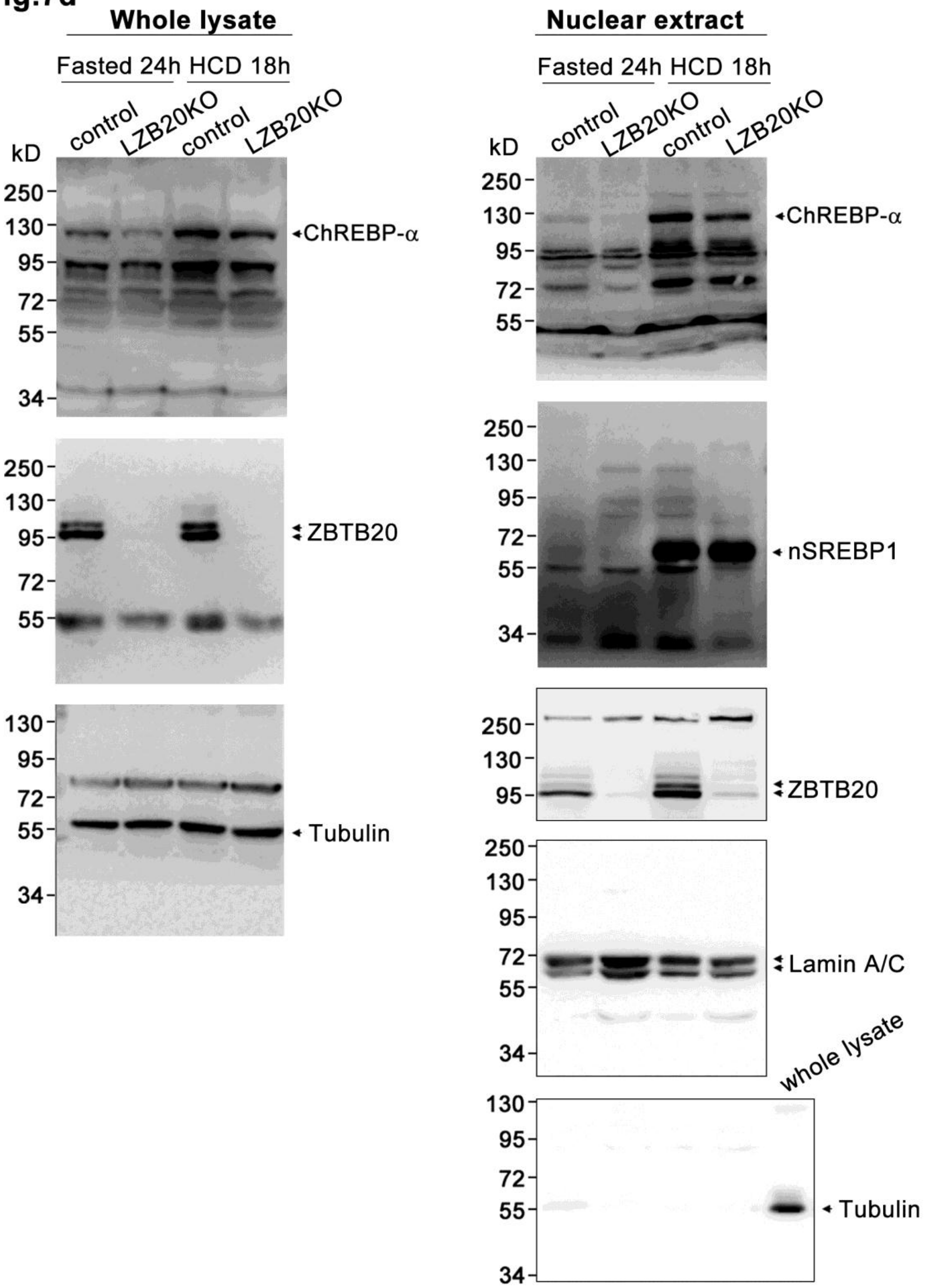
Whole lysate
Fasted 24h
HCD 18h
control
LZB20KO
control
LZB20KO
kD
250
130
95
72
55
34
ChREBP-α
ZBTB20
Tubulin
Nuclear extract
Fasted 24h
HCD 18h
nSREBP1
Lamin A/C
whole lysate

Fig. 8f

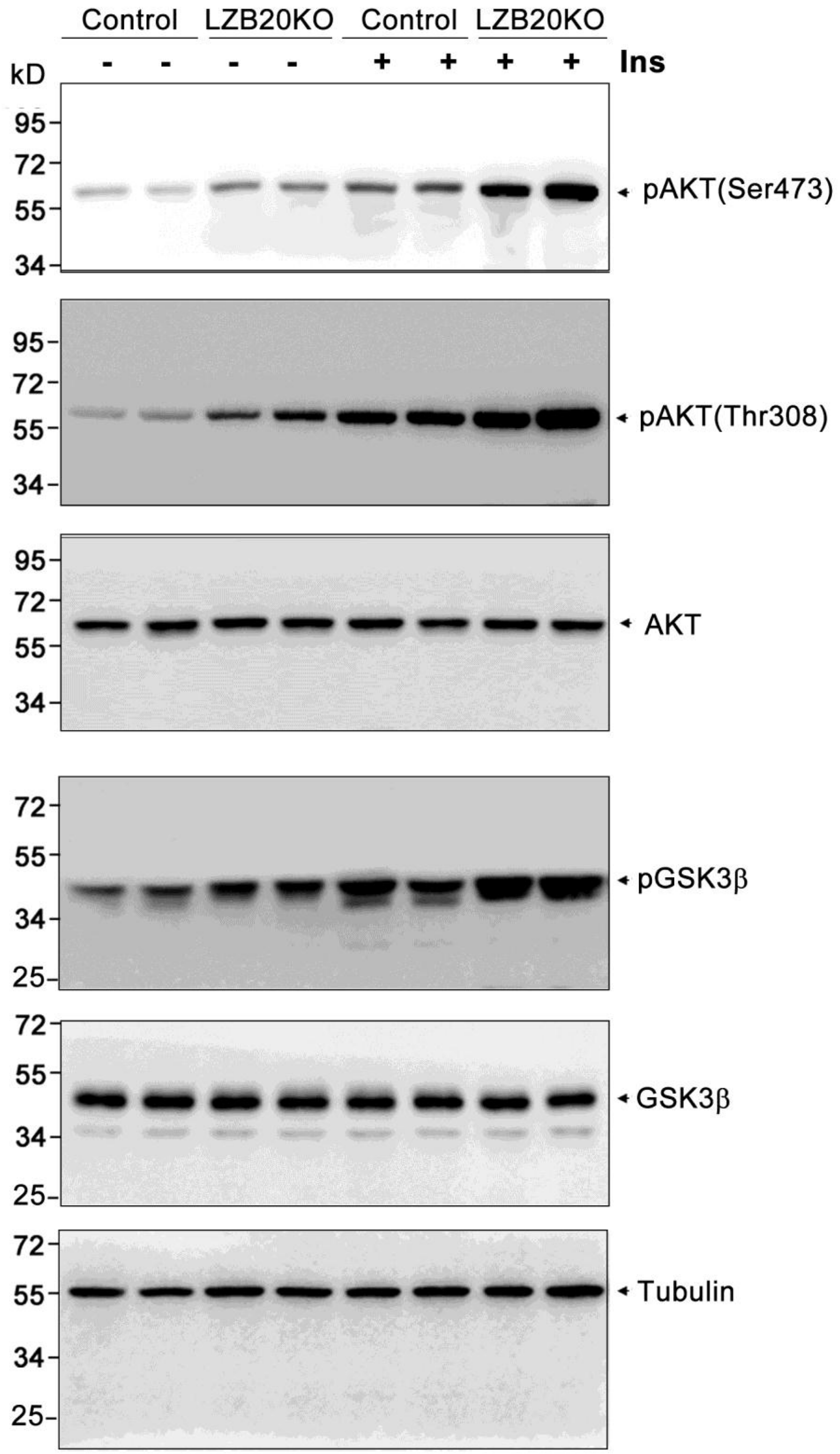

Supplementary Figure 13. Uncropped images of Western blots for figures 3 to 8. N.S., nonspecific bands.

Supplementary Table 1. Metabolic parameters under normal chow

Parameters	control	LZB20KO	n	t-test value
Body weight (g)	24.1±1.7	24.3±2.4	7	0.899
Liver weight (g)	1.05±0.09	1.00±0.14	7	0.434
Liver/body weight ratio（%）	4.4±0.35	4.3±0.29	7	0.163
Lean mass（%）	81.5±1.3	80.0±2.3	5	0.262
Fat mass（%）	7.3±1.2	8.5±2.4	5	0.334
Plasma total protein（g/L）	57.5±5.4	56.2±5.0	4	0.743
Plasma albumin（g/L）	24.3±2.1	23.6±3.3	4	0.748
Plasma apoB (mg/ml)	0.56±0.04	0.53±0.03	4	0.865
Plasma AKP（u/L）	171.0±29.5	157.5±16.8	4	0.457
Plasma ALT（u/L）	53.4±7.8	75.5±24.9	4	0.141
Blood glucose, Fasted (mmol/L)	3.45±0.31	3.70±0.21	5	0.528
Blood glucose, Fed (mmol/L)	8.38±0.48	6.46±0.38	5	0.014*
Plasma insulin, Fasted (ng/ml)	0.249±0.06	0.229±0.04	5	0.791
Plasma insulin, Fed (ng/ml)	1.03±0.24	1.02±0.28	5	0.980

Male mice, 3~4 months old. * P<0.05 vs control (Student's t-test).

Supplementary Table 2. Liver mRNA levels in LZB20KO mice fed chow or HCD

Gene	Normal chow	HCD
Zbtb20	0.047**	0.042**
Glut2	1.10	0.96
Glut5	0.22*	0.25*
Gck	1.20	0.89
Khk	0.79	0.91
Pklr	0.39*	0.36*
Pfk	1.13	0.96
Acl	1.08	1.13
Acc1	0.82	1.02
Acc2	1.03	0.82
Fasn	0.40*	0.58*
Elovl6	0.34*	0.26*
Scd1	0.40*	0.48*
Gpat1	0.78	1.10
Dgat2	0.93	0.88
Ldlr	1.32	ND
Hmgcr	1.49	ND
Hmgcs	2.53*	ND
ChREBPα	0.64**	0.52*
ChREBPβ	$0.08^{\#}$	0.28*
Mlxα	1.08	0.92
Mlxβ	0.89	1.03
Srebp1c	1.33	1.14
Srebp2	0.90	0.86

Male wild-type and LZB20KO mice were fed normal chow or HCD for 2 months before sacrifice. Liver mRNA levels were determined by real-time RT-PCR. The comparative cycle threshold method was used to determine mRNA levels in *LZB20KO* mice relative to age and diet-matched wild-type control, which are arbitrarily assigned a value of 1 for each mRNA species. *36B4* mRNA level was used as the invariant control. ND, not detected. n=6 per group. *, p<0.05; **, p<0.01; $^{\#}$, p<0.001 vs control (Student's t-test).

Supplementary Table 3. Sequence of the primers used for RT-PCR analysis

Genes	Primers	Sequence (5'-3')	PCR product (bp)
Zbtb20	Forward	gcagccggcagccccttcttc	410
	reverse	cgctcgccgctgccattctg	
ChREBPα	forward	cgacactcacccacctcttc	121
	reverse	ttgttcagccggatcttgtc	
ChREBPβ	forward	tctgcagatcgcgtggag	87
	reverse	cttgtcccggcatagcaac	
Mlx	forward	cctggggcctggggataga	145
	reverse	tggggaaggcagtaggaaacat	
Srebp1c	forward	tttcggctggggcattctcactcc	274
	reverse	gaaatctccccgggccaaatccag	
Srebp2	forward	gcgttctggagaccatgga	131
	reverse	acaaagttgctctgaaaacaaatca	
LXR	forward	gcaggaccagctccaagtag	125
	reverse	ggctcaccagcttcattagc	
FXR	forward	atggggatgttggctgaatgtttg	189
	reverse	ggtctgctggtctgccgtgagtt	
RXR	forward	cgacgggagatgcggcacagttac	356
	reverse	tcccccaccatgccatccagttta	
Pparg	forward	atcgcgtacggcaatggctttatc	220
	reverse	caatcccctcctgcaacttctcaa	
PGC1a	forward	tcaaggtccccaggcagtagat	312
	reverse	tgtccgcgttgtgtcaggtc	
FXR	forward	atggggatgttggctgaatgtttg	186
	reverse	ggtctgctggtctgccgtgagtt	
Acc1	forward	cttcggggtggttcttgggttgtg	331
	reverse	cctgcatccggcctggtgtg	
Acc2	forward	cggagggcacggtggagatta	230
	reverse	catgtggcccggggtgtcgtg	
Fasn	forward	atcctggaacgagaacacgatct	140
	reverse	agagacgtgtcactcctggactt	
Elovl6	forward	tgccatgttcatcaccttgt	84
	reverse	tgctgcatccagttgaagac	
Scd1	forward	tggggctgctaatctctgggtgta	169
	reverse	ggctttatctctggggtgggtttg	

Gpat	forward	tgctgcggaaaaactacggctacg	271
	reverse	ttgctggcggtgaagagaatgtgc	
Dgat2	forward	ggggccgatgggtccagaagaag	99
	reverse	ccagcccccaggtgtcagaggaga	
Glut2	forward	ttccttgggccttacgtgttcttc	232
	reverse	cctggtcggttcctcggttttag	
Glut5	forward	aggcggcgggcttcatctctgt	254
	reverse	tcgccgtccccaaagctctaccac	
Gluk	forward	ctgctactatgaagaccgccaa	62
	reverse	aggcgttgcagccgg	
Khk	forward	cccaccgccccgagtagtagacac	270
	reverse	cacacctgccggggaatgg	
Pfk	forward	atggcaaagctatcggtgtc	178
	reverse	acacagtcccatttggcttc	
Plkr	forward	ggacaaggggcgatgcaaagacag	253
	reverse	accccgaagcgcagatccaaaaga	
apoB	forward	ggcttctcccctgtataatgtcac	248
	reverse	cctgccagtcccaaagtcct	
Mttp	forward	cgcgagtctaaaacccgagtg	241
	reverse	ccctgcctgtagatagcctttcat	

Supplementary Table 4. Information for the antibodies

Antibody	Company	Catalogue	Working dilution*
Anti-ChREBP (M-300)	Santa Cruz	sc-33764	1:1000
Anti-ChREBP	Novus	NB400-135	1:2000
Anti-SREBP1	Santa Cruz	sc-367	1:1000
Anti-SREBP1	Santa Cruz	sc-365513	1:1000
Anti-SREBP1	Santa Cruz	sc-8984	1:1000
Anti-SREBP1	Abcam	ab28481	1:2000
Anti-SREBP2	BD	557037	1:2000
Anti-SREBP2	Santa Cruz	sc-5603	1:1000
Anti-MLX	Novus	NBP1-68176	1:2000
Anti-MLX	Proteintech	12042-1-AP	1:1000
Anti-Akt (pan) (40D4)	Cell Signaling	2920	1:1000
Anti-Akt	Cell Signaling	9272	1:1000
Anti-phospho-AKT(Ser473)	Cell Signaling	4060	1:1000
Anti-phospho-Akt (Thr308) (D25E6)	Cell Signaling	13038	1:1000
Anti-phospho-Akt (Thr308)	Cell Signaling	9275	1:1000
Anti-GSK-3β (3D10)	Cell Signaling	9832	1:1000
Anti-phospho-GSK-3β(Ser9) (5B3)	Cell Signaling	9323	1:1000
Anti-HMGCS1	GeneTex	GTX112346	1:1000
Anti-HMGCR	Novus	NBP1-5073	1:1000
Anti-FLAG M2	Abmart	M2008	1:2000
Anti-α-Tubulin	Sigma	T6074	1:4000
Anti-α-Tubulin	Proteintech	66031-1	1:5000
Anti-Lamin A/C	Cell Signaling	2032	1:2000
Anti-ZBTB20 (9A10)	Home-made		1:5000

*Working dilutions for western blotting

Supplementary Table 5. Sequence of the primers used for ChIP analysis

Genes	Orientation	Sequence (5'-3')	Position*	PCR product
ChREBPα	forward	tctgtggatcgtgaaccctattt	-2485	220 bp
	reverse	tttctcgcctgccctctagta	-2704	
ChREBPβ	forward	gggctggggctggggctgagact	-331	345 bp
	reverse	ccggcttttagactggggtgtgga	+14	
Glut5	forward	gagagccctggggatggatgtc	-277	485 bp
	reverse	CTTCGGGGGCGGGAGTGAGAG	+208	
Pklr	forward	gcacgcctttaatcccagcacttg	-604	467 bp
	reverse	cttcccctaggtcatccgtttttc	-138	
Fasn	forward	gtccctgcccgcatcctggtctcc	-293	390 bp
	reverse	AGTGTGGGTCGGGCGTGCTTGGTC	+97	
Elovl6	forward	ctgcgggatcgaggtagtgaggag	-414	346 bp
	reverse	gagagggcgcagaagagcaagacc	-69	
Scd1	forward	ccggggtttctctttgctggtg	-552	390 bp
	reverse	cctgcctggggataaatgctaatg	-163	

* Position relative to transcription start site (+1) in bp
